# Transistor‐Level Activation Functions via Two‐Gate Designs: From Analog Sigmoid and Gaussian Control to Real‐Time Hardware Demonstrations

**DOI:** 10.1002/adma.202511018

**Published:** 2025-11-24

**Authors:** Junhyung Cho, Youngmin Han, Won Woo Lee, Youngwoo Yoo, Kannan Udaya Mohanan, Chang‐Hyun Kim, Junhwan Choi, Young‐Joon Kim, Wonjun Shin, Hocheon Yoo

**Affiliations:** ^1^ Department of Artificial Intelligence Semiconductor Engineering Hanyang University 222 Wangsimni‐ro Seoul 04763 Republic of Korea; ^2^ Department of Electronic Engineering Hanyang University 222 Wangsimni‐ro Seoul 04763 Republic of Korea; ^3^ Department of Semiconductor Engineering Gachon University Seongnam‐si Gyeonggi‐do 13120 Republic of Korea; ^4^ School of Electrical Engineering and Computer Science University of Ottawa Ottawa ON K1N 6N5 Canada; ^5^ Department of Chemical Engineering Dankook University 152 Jukjeon‐ro, Suji‐gu Yongin Gyeonggi‐do 16890 Republic of Korea; ^6^ Department of Semiconductor Convergence Engineering Sungkyunkwan University Suwon 16419 Republic of Korea

**Keywords:** anti‐ambipolar transistor, Gaussian activation function, hardware application, multilayer perceptron, prediction of change, screen gate structure, sigmoid activation function

## Abstract

Tunable analog activation functions are essential for energy‐efficient artificial intelligence (AI) hardware. Two transistor designs are presented: the sigmoid‐like activation function transistor (SA‐transistor) and the Gaussian‐like activation function transistor (GA‐transistor), which implement analog sigmoid and Gaussian functions using a screen gate structure. In the SA‐transistor, adjusting the screen gate voltage (*V*_Screen‐G_) enables precise control of the sigmoid slope and saturation level. In the GA‐transistor, the amplitude and standard deviation of the Gaussian response are tunable through the same mechanism. These transistors enable precise and continuous tuning of analog activation parameters such as slope, amplitude, and width at the device level. This controllability allows hardware‐optimized neural computations tailored to specific tasks or datasets. Applied in real‐world tasks, the SA‐transistor improved lung magnetic resonance imaging (MRI) classification accuracy from 77% to 84%, and the GA‐transistor raised the time‐series forecasting coefficient of determination (*R* ^2^) from 0.82 to 0.93. Furthermore, by assembling these devices into a hardware‐based multilayer perceptron (MLP), robust inference is demonstrated on the IRIS dataset with 96.7% overall accuracy. This system‐level validation highlights that analog activation transistors can directly support neuromorphic accelerators without digital post‐processing, reducing circuit complexity and power consumption while maintaining high classification fidelity.

## Graphical abstract

Screen gate‐based transistors are presented, enabling tunable analog sigmoid and Gaussian activations. The SA‐transistor improves MRI classification accuracy, while the GA‐transistor supports precise Gaussian kernel tuning for forecasting. Both functions are implemented in a single device, offering compact, energy‐efficient analog AI processing solutions.

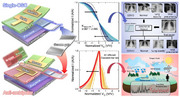

## 1 Introduction

Despite their ubiquity in neural networks, conventional software‐based implementations of activation functions are computationally intensive and power‐hungry.^[^ ^1^, ^2^, ^3^ ^]^ As neural networks continue to grow in complexity and application breadth, such as natural language processing, computer vision, and autonomous control, the need for real‐time, low‐power operation becomes increasingly important.^[^ ^4^, ^5^, ^6^ ^]^ Activation functions, particularly sigmoid^[^ ^7^, ^8^, ^9^ ^]^ and Gaussian^[^ ^10^, ^11^, ^12^ ^]^ types, are essential in enabling nonlinear transformations that underpin learning and decision‐making processes in these systems. However, realizing such functions in software often involves iterative arithmetic operations or large look‐up tables, leading to considerable computational overhead and energy consumption. These challenges are particularly acute in resource‐constrained environments such as edge computing and neuromorphic platforms, where efficiency and compactness are critical.^[^ ^13^, ^14^, ^15^ ^]^ Consequently, the limitations of conventional software‐based approaches pose a significant barrier to the broader integration of machine learning capabilities into hardware systems.

To address the limitations of software‐based implementations, activation functions have been emulated using conventional complementary metal‐oxide‐semiconductor (CMOS) circuitry. In these designs, nonlinear transfer characteristics are typically synthesized through a combination of analog components including differential pairs, current mirrors, and operational amplifiers.^[^ ^16^, ^17^, ^18^ ^]^ For example, sigmoid and Gaussian responses have been approximated using multi‐stage transistor configurations that incorporate feedback loops or piecewise linear segments. Although such circuits enable the on‐chip realization of nonlinear functions, these implementations often require multiple transistors and additional components, such as operational amplifiers, digital logic gates, which increase power consumption, circuit area, and design complexity.^[^ ^19^, ^20^ ^]^ These factors underscore the need for more compact device‐level approaches that exhibit intrinsic nonlinear behavior and allow for precise tunability of activation functions. While look‐up table (LUT) based implementations can be highly efficient in low‐precision scenarios due to their compactness and speed, they inherently store static activation curves and cannot adapt during training, limiting their adaptability.^[^ ^21^, ^22^ ^]^ An emerging strategy to overcome these limitations involves engineering nonlinear activation characteristics directly at the device level, rather than through circuit‐level synthesis.^[^ ^23^ ^]^ To implement a device that exhibits electrical characteristics resembling a nonlinear activation function, a variety of device designs, including those based on heterojunctions are required, using combinations of materials with different polarities, energy band structures, and surface properties, such as organic semiconductors,^[^ ^24^, ^25^, ^26^, ^27^ ^]^ metal oxides,^[^ ^28^, ^29^, ^30^, ^31^ ^]^ 2D materials,^[^ ^32^, ^33^, ^34^, ^35^, ^36^ ^]^ and polymers.^[^ ^37^, ^38^, ^39^, ^40^, ^41^, ^42^ ^]^ While prior studies have made substantial progress in tailoring device structures and material systems to enhance conventional transistor performance, for instance, by increasing carrier mobility or improving switching characteristics, most efforts have remained focusing on optimizing metrics relevant to digital or binary logic applications.^[^ ^43^, ^44^, ^45^, ^46^ ^]^ Overcoming this limitation calls for the development of unconventional device designs that emphasize tunable nonlinear responses rather than traditional performance metrics.

In this context, we introduce two transistor architectures that allow tunable analog realization of the sigmoid and Gaussian functions by means of a screen gate design. Although several previous studies have demonstrated device‐level sigmoid‐like activation functions, most of them offered only limited tunability.^[^ ^8^, ^47^, ^48^, ^49^ ^]^ In our study, we show that the sigmoid‐like activation function transistor (SA‐transistor) allows for independent control over key features of the sigmoid‐like function, including its threshold, slope, and output level, by adjusting the screen gate and drain voltages. Unlike conventional double‐transistor circuits, our approach achieves tunable sigmoid and Gaussian responses in a single channel through electrostatic carrier injection control by a screen gate, reducing device count and bias complexity. The SA‐transistor is based on a single organic semiconductor (OSC) channel, with the screen gate positioned directly beneath the channel on the source electrode. The applied screen gate voltage (*V*_Screen‐G_) allows for systematic and predictable control over the sigmoid function characteristics. The threshold position (*X*_0_) can be shifted over a range of −10.34 V (from −2.36 to −12.70 V) by sweeping *V*_Screen‐G_ from −1 to −5 V. The slope (*k*), which defines the transition sharpness, can be modulated between 1.677 and 0.248 A V^−1^ depending on the differential *V*_Screen‐G_. Moreover, the saturation current level (*L*) varies by a factor of ≈34, ranging from 33.8 nA to 1.15 µA, enabling precise control over the analog output range. The Gaussian‐like activation function transistor (GA‐transistor), based on an anti‐ambipolar heterojunction consisting of serially connected n‐type and p‐type semiconductors, exhibits a symmetric current profile characteristic of Gaussian activation. By tuning the *V*_Screen‐G_, the mean (*µ*) of the Gaussian response shifts systematically from −1.94 to −5.75 V. The amplitude (*A*) can be tuned from 0.02 µA to 0.35 µA, while the standard deviation (*σ*) expands from 1.22 to 3.57 V. This allows the Gaussian response to be customized for narrow or broad activation bandwidths. These quantitative results validate the ability of screen gate electrostatics to directly encode key nonlinear activation functions with high precision. The tunability across threshold position, slope, amplitude, and mean makes this architecture a versatile and low‐power hardware platform for analog implementation of neural network activation functions, suitable for compact, low‐power artificial intelligence (AI) inference hardware with enhanced integration capability. By applying the proposed sigmoid and Gaussian activation transistors to lung magnetic resonance imaging (MRI) classification and environmental time‐series forecasting, respectively, we validated their practical utility in task‐specific scenarios. In a lung MRI classification task, the tunable sigmoid activation enabled by the SA‐transistor demonstrated an improvement by a factor of 1.09 over fixed activation functions, increasing the classification accuracy from 77% to 84%. This improvement is attributed to the ability of the SA‐transistor to adjust the slope of the activation function at the hardware level, which enhanced sensitivity to subtle grayscale variations in MRI images and led to more precise feature extraction. The GA‐transistor allowed tunable Gaussian shaping, facilitating precise temporal weighting in forecasting tasks. These results demonstrate the scalability and adaptability of the activation transistors for efficient neuromorphic hardware applications.

## 2 Results and Discussion

**Figure 1:**
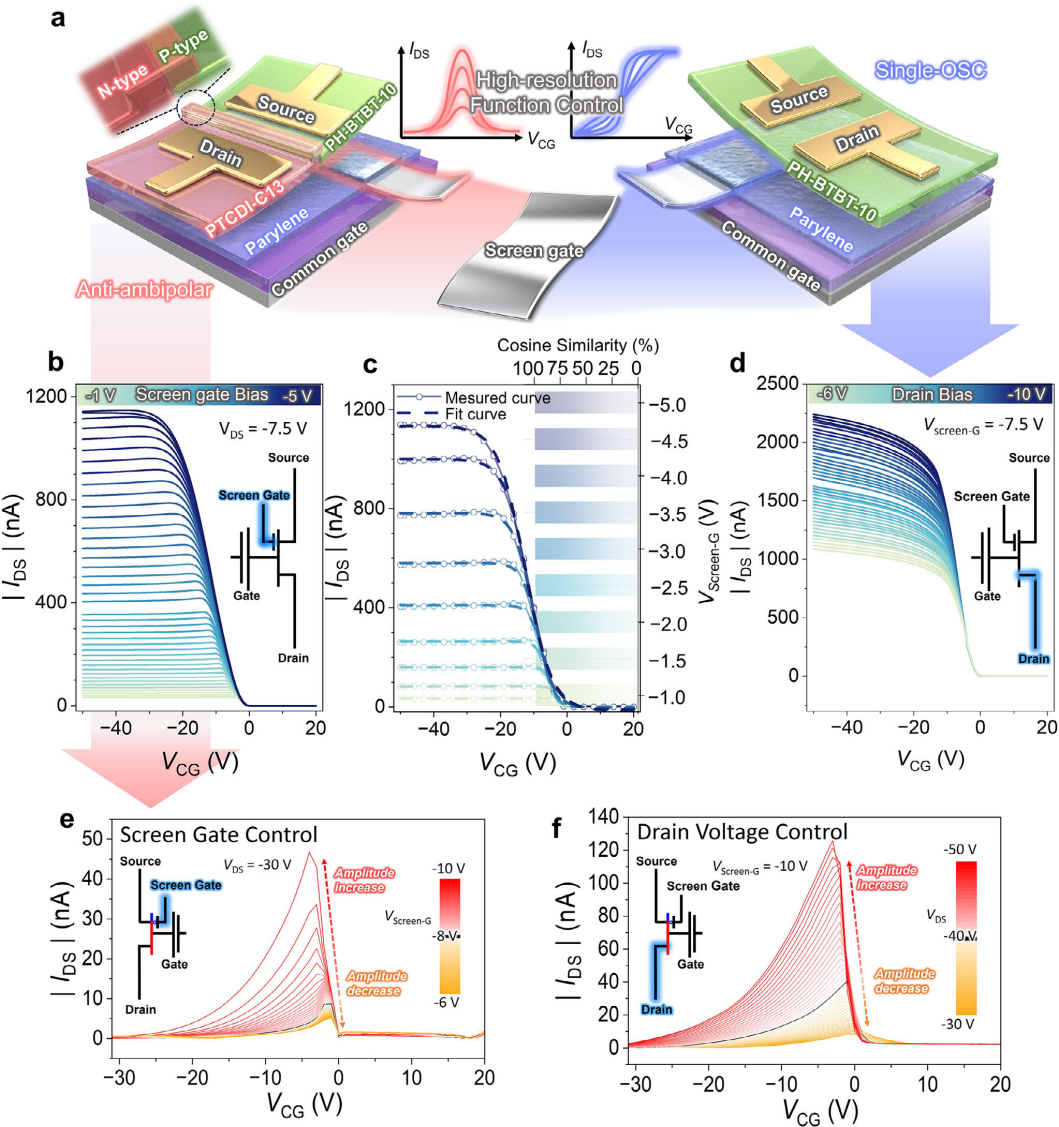
Schematic illustration of an inserted screen gate in organic semiconductor‐based devices and key electrical characteristics emulating activation function; a) Schematic overview of screen gate modulation applied to two types of device: a single‐channel PH‐BTBT‐10 transistor and a PTCDI‐C13/PH‐BTBT‐10 heterojunction device exhibiting anti‐ambipolar behavior. b) Screen gate‐controlled transfer characteristics of the SA‐transistor, obtained at a constant *V*_DS_ = −7.5 V within the *V*_Screen‐G_ range of −1–−5 V. c) Sigmoid function fitting result of transfer characteristics and cosine similarity across varying screen gate voltages (*V*_Screen‐G_ = −1 to −5 V). d) Drain bias‐controlled transfer characteristics, obtained at a constant *V*_Screen‐G_ = −7.5 V within the *V*_DS_ range of −6–−10 V. e) Screen gate‐controlled transfer characteristics of the GA‐transistor, obtained at a constant *V*_DS_ = −30 V within the *V*_Screen‐G_ range of −6–−10 V. f) Drain bias‐controlled transfer characteristics, obtained at a constant *V*_Screen‐G_ = −10 V within the *V*_DS_ range of −30–−50 V.

We designed two specialized transistor architectures. **Figure** **1** presents the structural concept (Figure 1a) and electrical characteristics of the SA‐transistors (Figure 1b–d) and GA‐transistors (Figure 1e,f), which are designed to implement sigmoid and Gaussian activation functions, respectively. In the SA‐transistor and GA‐transistor, the screen gate is asymmetrically positioned under the source‐side channel region, allowing independent control of device operation through the application of both a common gate voltage (*V*_CG_) and *V*_Screen‐G_. Unlike previous studies where split gates were primarily used to realize on/off switching,^[^ ^44^, ^50^ ^]^ our design incorporates a control gate and an asymmetric screen gate, both of which electrostatically modulate the same channel region. This asymmetric configuration enables precise modulation of the electrical characteristics of the transistors, making them a promising hardware‐level platform for tunable sigmoid and Gaussian activations. Figure 1a illustrates the device structures of the SA‐transistor, based on a PH‐BTBT‐10 p‐type single‐channel configuration, and the GA‐transistor, which employs an anti‐ambipolar heterojunction composed of PH‐BTBT‐10 and PTCDI‐C13. A distinctive commonality of both devices is the incorporation of a screen gate in the bottom‐gate, top‐contact configuration, enabling precise modulation of the channel behavior. Figure S1 (Supporting Information) visually presents the step‐by‐step fabrication procedures of both the SA‐transistor and GA‐transistor using optical microscopy (OM) images. The device dimensions, including channel length and width, are also provided. The deposition thicknesses of each active material and their annealing conditions are described in detail in the Experimental Section. Atomic force microscope (AFM) analysis (Figure S2, Supporting Information) confirms that the step heights of the fabricated devices were consistent with the experimental values. In terms of structure, the cross‐sectional scanning electron microscopy (SEM) image of the screen‐gate architecture (Figure S3, Supporting Information) and the surface SEM image of the anti‐ambipolar heterojunction region in the GA‐transistor (Figure S4, Supporting Information) demonstrate that the device structures were fabricated as designed. For elemental verification, element‐resolved SEM energy‐dispersive X‐ray spectroscopy (EDS) mapping (Figure S5, Supporting Information) provided the elemental composition of the devices and distinguished the patterned functional layers according to their elemental ratios for both the SA‐ and GA‐transistors.

To investigate the influence of *V*_Screen‐G_ on the electrical characteristics of the SA‐transistor, we measured and compared the transfer characteristics of a single‐gate transistor and an SA‐transistor incorporating a screen gate. While the single‐gate transistor exhibited a linear increase in current, the SA‐transistor showed a more distinctly defined sigmoid‐like behavior due to the current‐limiting effect of the screen gate (Figure S6, Supporting Information). The observed current‐limiting behavior in the saturation region demonstrated that the sigmoid‐like characteristics can be tuned via the screen gate. Subsequently, to quantitatively verify the controllability of the SA‐ transistor's electrical characteristics by the screen gate, we fixed *V*_DS_ at −5, −7.5, and −10 V, swept *V*_CG_ from +20 to −50 V in 1 V increments, and varied *V*_Screen‐G_ from −1 to −5 V in −0.1 V steps while measuring the transfer characteristics (Figure S7, Supporting Information). As shown in Figure 1b, under these measurement conditions, the most notable sigmoid‐like transfer characteristics were observed at *V*_DS_ = −7.5 V. This optimal behavior is attributed to the balanced carrier injection induced across the channel, which enables sufficient carrier injection while still preserving a gradual transition between off‐ and on‐states. Compared to the condition at *V*_DS_ = −7.5 V, a lower carrier injection at *V*_DS_ = −5 V results in reduced current levels, leading to a gentler slope in the transfer characteristics, as shown in Figure S7a (Supporting Information). In contrast, at *V*_DS_ = −10 V, the increased carrier injection causes the channel to saturate prematurely, thereby narrowing the effective transition region and suppressing the curvature of the sigmoid function, as shown in Figure S7b (Supporting Information). Among the tested conditions, *V*_DS_ = −7.5 V yielded the best agreement between the experimental data and the ideal sigmoid fitting, suggesting that this voltage range is optimal for achieving a well‐defined and tunable nonlinear response.

Figure 1c shows a comparison between the experimental data, which were extracted at 0.5 V intervals within the *V*_Screen‐G_ range of −1 to −5 V, and the theoretical logistic fitting curves derived from the model. These measurements were performed under a fixed *V*_DS_ of −7.5 V, and additional results obtained at *V*_DS_ values of −5 and −10 V are provided in Figure S8 (Supporting Information) for comparison. The measured and fitted transfer curves exhibit over 98.9% cosine similarity at *V*_DS_ of −7.5 V, validating the accurate modulation of sigmoid behavior and demonstrating a consistent relationship between analog activation characteristics and similarity‐based evaluation. As shown in Figure 1d, to compare the transfer characteristics under individual modulation of the *V*_DS_ and *V*_Screen‐G_, the transfer characteristics were measured while applying the same fixed voltage of −7.5 V to the *V*_Screen‐G_. As a result, as the *V*_DS_ increased, the number of injected carriers increased, resulting in a linear increase in drain‐source current (*I*_DS_) from 1.088 to 2.24 µA. Transfer characteristics were additionally obtained under fixed *V*_Screen‐G_ = −5 and −10 V (Figure S9, Supporting Information), increasing *V*_Screen‐G_ led to a higher *I*_DS_. Figure S10 (Supporting Information) presents the experimentally measured data points and the corresponding theoretical logistic fitting curves derived from the model, extracted at 0.5 V intervals within the *V*_DS_ range of −6–−10 V, under three different *V*_Screen‐G_ conditions: −5, −7.5, and −10 V. The cosine similarity analysis was conducted under a fixed *V*_Screen‐G_ of −7.5 V across the *V*_DS_ range of −6–−10 V, and the results are shown in Figure S11 (Supporting Information). All calculated cosine similarity values exceeded 99.3%, confirming a consistently high agreement between the measured transfer curves and the logistic fitting model under these conditions. The experimental transfer characteristics shown in Figure 1b,d demonstrates how the sigmoid behavior can be precisely modulated by adjusting *V*_Screen‐G_ and *V*_DS_, respectively.

Now we investigate the electrical characteristics of the GA‐transistor whose structure is shown at the left side of Figure 1a. To investigate the influence of the *V*_Screen‐G_ on the electrical characteristics of the GA‐transistor, *V*_DS_ was fixed at −10 V, and the electrical characteristics of the GA‐transistor were measured by varying *V*_Screen‐G_ from −6 to −10 V. As shown in Figure 1e, this configuration enables the evaluation of how variations in *V*_Screen‐G_ modulate the transfer characteristics of the device. Specifically, as *V*_Screen‐G_ was increased from −6 to −10 V, corresponding to the application of a stronger negative bias, peak voltage (*V*_peak_) exhibited a systematic negative shift from −1 to −4 V, while maximum drain current (*I*_peak_) was precisely tuned from 5.14 to 46.75 nA. Conversely, to observe the influence of *V*_DS_ on the transfer characteristics, *V*_DS_ was swept from −30 to −50 V in −0.5 V steps while maintaining *V*_Screen‐G_ at −10 V. As shown in Figure 1f, the *I*_peak_ increased monotonically with *V*_DS_, indicating enhanced carrier injection and under stronger drain bias conditions. While the overall anti‐ambipolar behavior remains, higher *V*_DS_ results in increased current levels and a more pronounced peak, which improves the signal amplitude available for Gaussian tuning. When *V*_DS_ was set to −30 V, the transfer curve exhibited *I*_peak_ of 8.2 nA at *V*_peak_ of 0 V. Upon increasing *V*_DS_ to −50 V, *V*_peak_ shifted negatively by 3 to −3 V, and *I*_peak_ increased significantly to 125.8 nA. As *V*_DS_ increases, carrier injection is enhanced, leading to a systematic shift of the *V*_peak_ toward more negative values and a significant increase in the *I*_peak_. By enabling effective tuning of the Gaussian response even at lower *V*_DS_ operating ranges, the *V*_Screen‐G_ control serves as a powerful control terminal for bias‐efficient modulation. The proposed dual‐voltage tuning approach enables hardware‐level implementation of sigmoid‐like and Gaussian‐like activation functions solely through gate architecture and electrostatic control, without the need for complex peripheral circuitry. In addition, to further emphasize the characteristics of the proposed approach, we investigated a comparative summary of previous studies that demonstrated sigmoid‐like and Gaussian‐like electrical responses at the device level (**Tables** **1** and **2**). As shown in Tables 1 and 2, prior works typically relied on multi‐material heterostructures or interconnected multiple devices, which often entail additional fabrication steps and structural complexity. In contrast, our device achieved tunable sigmoid‐like characteristics simply by incorporating a screen gate into a single semiconductor channel, thereby demonstrating both structural simplicity and the novelty of the proposed concept.

**Table 1:** Summary of previous works implementing and modulating sigmoid and Gaussian‐like functions at the device level.

	Sigmoid‐like characteristics device						
Year	Device type	Material	Device number	Sigmoid modulation method	Application	On‐board integration	Refs.
2024	Heterojunction transistor	CIPS/MoS_2_	1	Tunneling barrier control	Face image classification	X	[8]
2021	Homogeneous transistor memory	WeS_2_/LiNbO_3_	12	Ferroelectric polarization control	Letter pattern classification	X	[47]
2023	Floating Gate Transistor	MoS_2_/hBN/Graphene	1	Floating gate accumulation control	Handwritten digit recognition	X	[48]
2021	Spintronic device	L1_1_‐CuPt/CoPt	1	Spin‐orbit torque control	Handwritten digit recognition	X	[49]
2025	Screen gate transistor	PH‐BTBT‐10	1	Screen gate bias control	MRI image classification	O	This work
	Gaussian‐like characteristics device						
Year	Semiconducting material type	Material	Electrode number	Operation voltage range	Application	On‐board integration	Refs.
2019	2‐Dimension	BP/MoS_2_	6	−50 to 50 V	Brainwave Recognition	X	[12]
2019	2‐Dimension	MoS_2_/MoTe_2_	3 + laser	−50 to 40 V	N/A	X	[51]
2018	Organic	DPA/CMUT	3 + light	−50 to −10 V	N/A	X	[52]
2017	2D/Organic	MoS_2_/Pentacene	3	−70 to −10 V	N/A	X	[53]
2025	Organic	PH‐BTBT‐10/PTCDI‐C13	4	−30 to 10 V	Forecasting prediction	O	This work

**Table 2:** Summary of previous works implementing and modulating sigmoid and Gaussian‐like functions at the device level.

Activation function	Device structure	Gate structure	Device simulation	Flexibility	On‐board integration	Refs.
Gaussian	Anti‐ambipolar heterojunction	Single gate	Electron and hole distribution	X	X	[44]
Negative trans‐conductance	Anti‐ambipolar heterojunction	Single gate	SPICE simulation	X	X	[50]
Gaussian	Dual anti‐ambipolar heterojunction	Single gate	Theoretical simulation	X	X	[54]
Sigmoid	Single channel	Single gate	COMSOL multiphysics simulation	X	X	[55]
X	Ambipolar heterojunction	Top split gate	X	X	X	[56]
Sigmoid/Gaussain	Single/Anti‐ambipolar heterojunction	Screen gate	Drift‐diffusion simulation	O	O	This work

**Figure 2:**
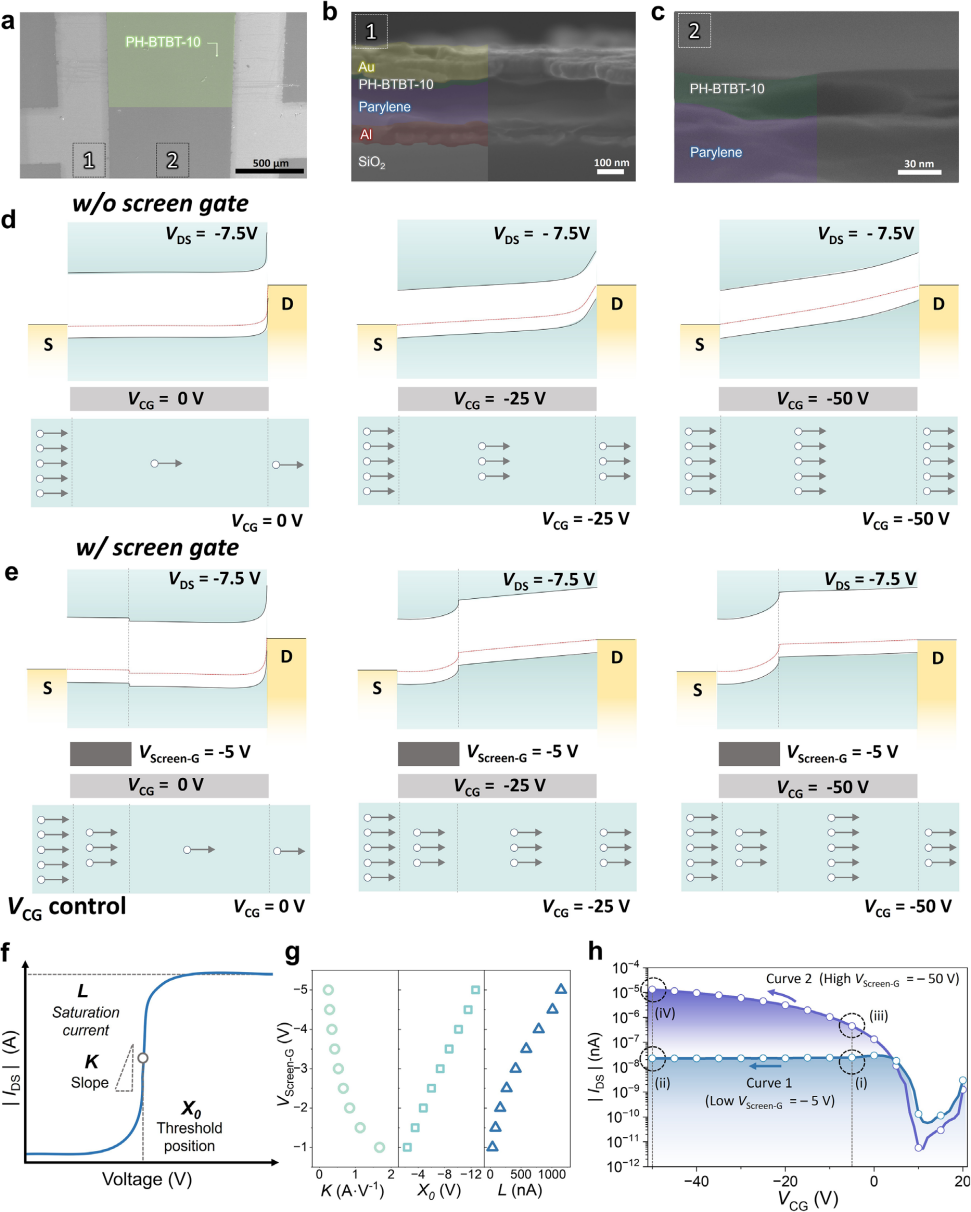
SEM images measured to confirm the structure of the SA‐transistor: a) surface SEM image, b) overall cross‐sectional SEM image, and c) detailed cross‐sectional SEM image between the heterojunction and dielectric layers. Energy band diagram for comparison of carrier transport illustration. The *V*_DS_ is applied −7.5 V, and *V*_CG_ is 0, −25, and −50 V, respectively. d) single‐channel transistor without a screen gate. e) single‐channel transistor with screen gate (SA‐transistor). f) Illustration of sigmoid function parameters; *L*: saturation current, *k*: slope, *X*_0_: threshold position. g) Extracted sigmoid parameters (*k*, *X*_0_, *L*) as a function of screen gate voltage. h) Two representative transfer curves (Curve 1 and Curve 2) are presented to illustrate the operating mechanism of sigmoid‐shaped transfer behavior in the SA‐transistor. Four distinct regions (i–iv) are defined based on the combinations of *V*_Screen‐G_ and *V*_CG_: (i) low *V*_Screen‐G_, *V*_CG_; (ii) low *V*_Screen‐G_, high *V*_CG_; (iii) high *V*_Screen‐G_, low *V*_CG_; and (iv) high *V*_Screen‐G_, *V*_CG_. Here, low and high bias conditions correspond to –5 and –50 V, respectively.

**Figure** **2**a shows the surface SEM image of the SA‐transistor, which previously demonstrated sigmoid behavior. The cross‐sectional SEM images (Figure 2b,c) were obtained by observing regions both with and without the inserted screen gate. These cross‐sectional views confirm the successful fabrication of the device, specifically illustrating the integration of the screen gate near the source electrode. This placement is consistent with the design objective of utilizing the screen gate to control carrier injection into the channel. To establish the physical characteristics of the device with and without a screen gate, the energy band diagrams of SA‐transistors are presented. Figure 2d illustrates the energy band modulation of a single p‐type transistor without a screen gate as a function of *V*_CG_. At *V*_CG_ = 0 V, the hole concentration in the channel is relatively low; however, due to the negative *V*_DS_, the band profile rises sharply, leading to a pronounced pinch‐off phenomenon. As *V*_CG_ increases further negatively to −25 and −50 V, the hole concentration in the p‐type channel increases, resulting in a progressively smoother band slope between the source and drain. In other words, as *V*_CG_ is increased in magnitude, hole accumulation monotonically increases across the entire source–drain channel, leading to a continuous rise in *I*_DS_. In contrast, Figure 2e shows that in the presence of a screen gate, the region beneath the screen gate is pre‐charged with holes by *V*_Screen‐G_. Consequently, this region is less sensitive to variations in *V*_CG_, exhibiting a sigmoidal response in the energy band profile and the associated current characteristics.

To analyze the sigmoid activation behavior of the proposed device, we first examine its sigmoid transfer characteristics. Figure 2f provides a conceptual illustration of the sigmoid function used to describe the transfer characteristics of the SA‐transistor structure. It highlights the key parameters of the logistic model such as threshold position (*X*_0_), saturation current level (*L*), and slope (*k*), which define the shape and behavior of the sigmoid response.^[^ ^57^, ^58^, ^59^ ^]^

(1) $$I\left(V\right)=\frac{L}{1+e^{-k\left(V-X_{0}\right)}}$$

In the context of the transfer characteristics, *X*_0_ represents the voltage where the transfer curves most steeply transition, functioning similarly to a threshold voltage (*V*_th_) and indicating the start of significant carrier injection. Parameter *L* represents the maximum current achievable when the transistor is fully activated, with higher values indicating improved carrier transport capability. Parameter *k* controls the slope of the sigmoid curve, dictating how sharply the current transitions from off‐state to the on‐state; a higher *k* value results in a sharper transition, whereas a lower *k* value indicates a more gradual transition. Collectively, the parameters *X*_0_, *L*, and *k* define the overall shape of the sigmoid function and serve as critical descriptors for evaluating how closely the experimentally obtained transfer characteristics replicate the expected sigmoid behavior. The values of *X*_0_, *L*, and *k* vary systematically with the applied *V*_Screen‐G_, indicating that the screen gate serves as an effective means to control the activation behavior of the transistor.

To examine how the *V*_Screen‐G_ influences the activation behavior of the transistor, we analyzed the variation of the sigmoid parameters *X_0_*, *L*, and *k* as a function of *V*_Screen‐G_. This analysis reveals how the screen gate electrostatics directly modulate the threshold position, saturation current, and slope of the transfer characteristics. Figure 2g examines how the sigmoid parameters *X*_0_, *L*, and *k* vary with changes in *V*_Screen‐G_, while the corresponding analysis with respect to *V*_DS_ is presented in Figure S12 (Supporting Information). As *V*_Screen‐G_ is swept from −1 to −5 V under a fixed *V*_DS_ of −7.5 V, *X*_0_ shifts toward more negative values, increasing from −2.36 to −12.70 V. This is because a more negative *V*_Screen‐G_ enhances carrier injection near the source‐side channel, leading to higher current levels and a corresponding increase in *X*_0_. As a result, the entire sigmoid curve moves in the negative direction along the *V*_CG_ axis. At the same time, the *L* value increases from 3.38 × 10^−8^ A to 1.15 × 10^−6^ A, indicating that the on‐state current becomes larger as more carriers accumulate in the channel. Additionally, the *k* value decreases from 1.677 to 0.248 A V^−1^ as *V*_Screen‐G_ becomes more negative, indicating a broadening of the transition region from the off state to the on state. At a relatively small *V*_Screen‐G_ of −1 V, the low saturation current level enables *I*_DS_ to reach its maximum value over a narrow *V*_CG_ range, leading to a steep transition and a large *k* value. However, at a larger negative *V*_Screen‐G_ of −5 V, the slope within the initial gate range (*V*_CG_ = 0 to −5 V) remains nearly identical to the −1 V case. Within the *V*_CG_ range of −5–−50 V, the channel region beneath the non‐screen gate becomes strongly accumulated, and a weak current starts to flow through the screen‐gated region. This gradual onset of current delays the saturation points to more negative *V*_CG_ values, resulting in a broader transition region and a more gradual slope in the sigmoid transfer curve, which in turn reduces the overall *k* value. When *V*_Screen‐G_ was fixed at −7.5 V, increasing *V*_DS_ from −6 to −10 V increased L from 1.07 to 1.27 µA and *k* from 0.17 to 0.3 A V^−1^, while *X*_0_ shifted from −15.19 to −11.55 V (Figure S13, Supporting Information). The higher drain bias enhanced carrier injection by lowering the energy barrier, which promoted stronger carrier transport. This led to a higher on‐state current (increase in *L*), a steeper slope in the turn‐on region (increase in *k*), and an earlier turn‐on closer to 0 V (shift of *X*_0_ toward the positive direction). In contrast, when *V*_DS_ was fixed at −7.5 V, increasing *V*_Screen‐G_ from −1 to −5 V increased *L* from 1.1 to 2.76 µA, but decreased *k* from 0.54 to 0.3 A V^−1^, and shifted *X*_0_ from −6.41 to −9.33 V (Figure S14, Supporting Information). The more negative screen gate bias induced stronger hole accumulation in the channel, which increased the on‐state current (increase in *L*) but also allowed residual conduction in the off current region. As a result, the device exhibited a gradual current increase instead of a sharp turn‐on, reducing the slope in the transition region (decrease in *k*). Furthermore, because the onset of steep current growth was delayed by this gradual turn‐on behavior, a larger negative *V*_CG_ was required to reach the rapid increase region, shifting *X*_0_ to more negative voltages.

A detailed investigation into the screen gate modulation reveals that the underlying mechanism originates from the interaction between screen gate control and electrostatic screening effects. To clarify the mechanism by which the transfer characteristics are modulated by the respective magnitudes of the *V*_CG_ and *V*_Screen‐G_, two representative transfer curves were extracted, as shown in Figure 2h. In this analysis, the *V*_DS_ was fixed at −5 V, *V*_Screen‐G_ was set to −5 and −50 V, respectively, and *V*_CG_ was commonly swept from 20 to −50 V. The current modulation behavior presented in Figure 2h provides direct electrical validation of the energy‐band evolution illustrated in Figure 2d,e. The device operation can be divided into four regions (i, ii, iii, iv) according to the bias combinations of *V*_CG_ and *V*_Screen‐G_. In region (i) (weak *V*_CG_ and *V*_Screen‐G_), the screen gate partially shields the source‐side channel, as predicted in Figure 2b, resulting in limited hole injection and suppressed *I*_DS_. In region (ii), although *V*_CG_ is highly negative, the applied *V*_Screen‐G_ of −5 V induces a pronounced screening effect that restricts carrier injection. Consequently, the drain current remains saturated even under a large *V*_CG_, serving as experimental evidence of the flattened energy‐band region beneath the screen gate, as predicted by the band diagram. As *V*_Screen‐G_ becomes more negative (region iii), the previously depleted region under the screen gate transitions into a stronger accumulation state. The enhanced hole accumulation enhances carrier injection into the channel, leading to an increase in *I*_DS_. In region (iv), both *V*_CG_ and *V*_Screen‐G_ are strongly negative, and the enhanced carrier transport yields a clear increase in *I*_DS_ along the channel. The resulting enhancement in the current experimentally verifies the dominant screening effect of the screen gate, confirming that current modulation is primarily governed by the electrostatic control from *V*_Screen‐G_. These bias‐dependent transitions quantitatively demonstrated the band‐modulation mechanism, established that the tunable transfer behavior originated from the electrostatic coupling between the screen gate and the common gate. In addition, we analyzed device physics through low‐frequency noise measurements, which provide direct evidence of the bias‐dependent transport mechanisms underlying the observed sigmoid‐like and Gaussian‐like behaviors (see Experimental section for detailed low‐frequency noise analysis of GA‐ and SA‐ transistors).

Moreover, to clarify the effect of the *V*_Screen‐G_, we first performed drift−diffusion simulations on a single‐channel transistor. Figure S15a (Supporting Information) shows that the key features of the transfer characteristics, including the *V*_Screen‐G_ control (measured while varying *V*_Screen‐G_ from −5 to −1 V), are well reproduced by our simulations, confirming that the experimentally observed behaviors are theoretically justifiable and predictable. Figure S15b (Supporting Information) compares the transfer curves for devices with and without a screen gate. While the device without a screen gate does not exhibit saturation, the strong saturation of *I*_DS_ when *V*_CG_ is biased more negatively (i.e., the sigmoid‐like curve) in the device with a screen gate originates from the screen effect of the screen gate. To further understand this difference, we calculated the hole concentration inside the semiconductor layer at *V*_CG_ = 0, −25, and −50 V. For the no‐screen‐gate device in Figure S15c (Supporting Information), when *V*_CG_ is biased more negatively, hole accumulation along the entire source−drain channel increases monotonically, leading to a constant rise in *I*_DS_. However, in the screen‐gate device, even when *V*_CG_ is biased at a larger negative voltage, the region above the screen gate in Figure S15d (Supporting Information) is pre‐charged with holes by *V*_Screen‐G_ and remains largely unaffected, producing the sigmoid‐like transfer behavior. As a result, through the consistency between the mechanism inferred from electrical characteristics and that obtained from drift–diffusion simulations, we demonstrated evidence that supported our explanation for the sigmoid‐like behavior and controllability induced by the screen gate. From these 2D hole distributions, we extracted 1D potential profiles in Figure S16 (Supporting Information) at 1 nm above the PH‐BTBT‐10/parylene interface to capture carrier accumulation and surface transport. In Figure S16a (Supporting Information), the no‐screen‐gate device, increasing *V*_CG_ from 0 to −50 V yields an almost linear potential drop along the channel, reflecting a uniform carrier distribution. By contrast, the SA‐transistor in Figure S16b (Supporting Information), the screen‐gate‐controlled region (x = 200 to 500 µm) maintains a constant resistance set by *V*_Screen‐G_, while the resistance of the common‐gate‐controlled region (x = 500 to 1200 µm) decreases as *V*_CG_ is biased more negatively. This redistribution of channel resistance causes the more resistive screen‐gate region to hold most of *V*_DS_ at *V*_CG_ = −50 V.

To further investigate the structural aspect of the SA‐transistor, we carried out additional simulations involving two types of structural variables, namely the position of the screen gate and the extension length (*L*_ext_) of the screen gate. Figure S17a (Supporting Information) visualizes two positions of the screen gate considered. Figure S17b (Supporting Information) confirms that the position of the screen gate significantly affects the transfer curve and that the sigmoid‐like feature only arises when the source is coupled with the screen gate. When *V*_Screen‐G_ is applied under the drain, there is no pre‐accumulation by the screen gate as *V*_DS_ <*V*_Screen‐G_, and the hole density can be controlled by *V*_CG_ over a wider region. Figure S17c (Supporting Information) graphically defines *L*_ext_, which would be a critical structural parameter given the mechanistic understanding obtained so far. Figure S17d (Supporting Information) shows that increasing *L*_ext_ decreases the saturated level of *I*_DS_ without affecting much the low *V*_CG_ regime. This is because the pre‐charged holes will contribute to the initial increase in *I*_DS_ while limiting the maximum channel conductivity when *L*_ext_ becomes too high.

**Figure 3:**
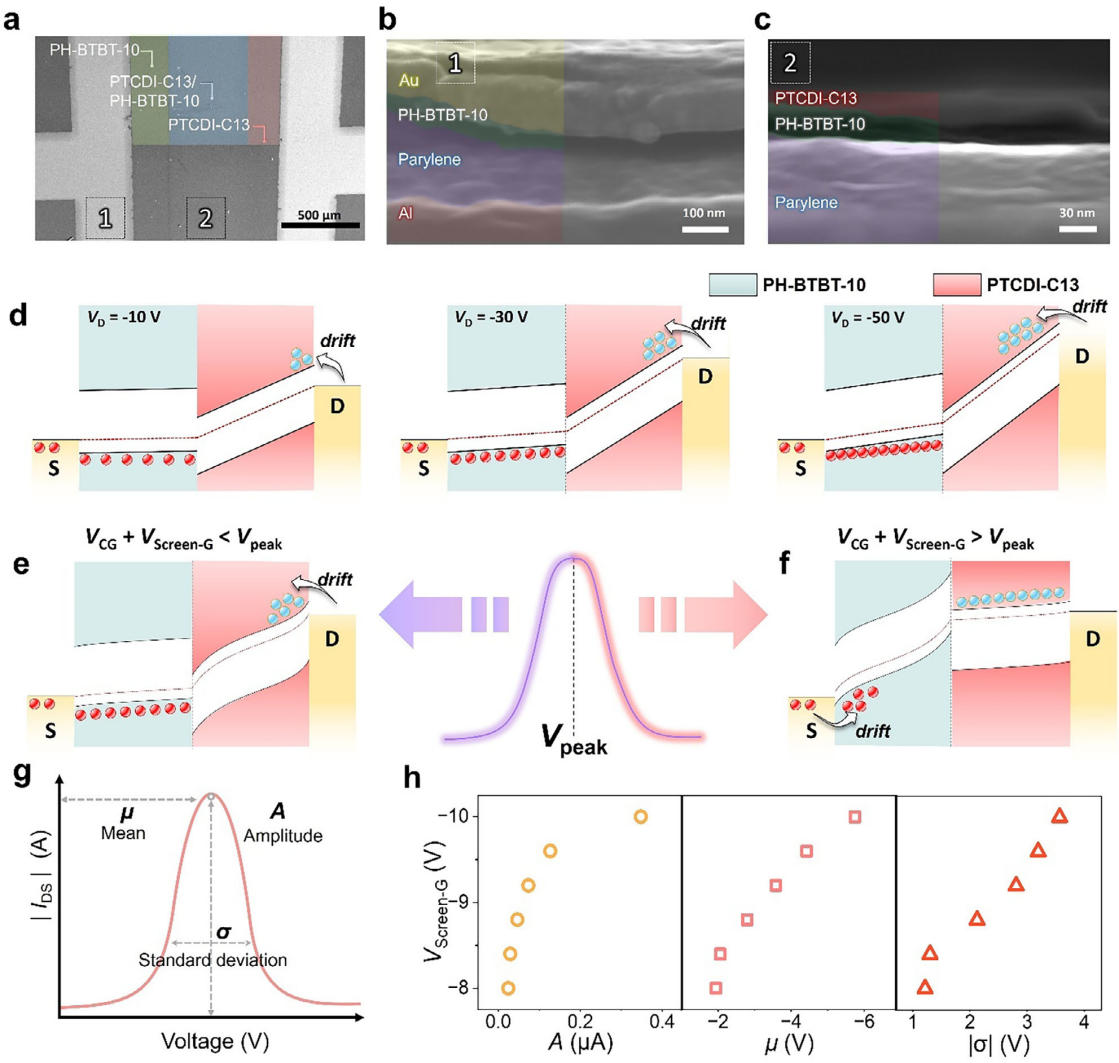
SEM images measured to confirm the structure of the GA‐transistor: a) surface SEM image, b) overall cross‐sectional SEM image, and c) detailed cross‐sectional SEM image between the heterojunction and dielectric layers. Energy band diagram for comparison of carrier transport illustration. d) Energy‐band diagrams of the pn heterojunction under constant *V*_Screen‐G_ and *V*_CG_ with *V*_DS_ varied from −10 to −50 V. And energy‐band diagrams illustrating the origin of the Gaussian‐shaped transfer characteristics. e) For *V*_CG_ + *V*_Screen‐G_ < *V*_peak_, the p‐channel exhibits a weaker field while the n‐channel shows a stronger field, leading to current increase. f) For *V*_CG_ + *V*_Screen‐G_ >*V*_peak_, the field distribution is reversed, resulting in a current decrease. g) Illustration of Gaussian function parameters; *A*: amplitude, μ: mean, σ: standard deviation. h) Extracted Gaussian parameters (*A*, μ, σ) as a function of *V*_Screen‐G_.

We next examine the GA‐transistor to highlight the distinct modulation behavior under similar biasing conditions. The SEM surface image (**Figure** **3**a) confirms the successful formation of the PH‐BTBT‐10 and PTCDI‐C13 heterojunction, exhibiting a clear interface between the two materials positioned between the electrodes. Furthermore, the cross‐sectional view in Figure 3b reveals the screen gate to be distinctly and well‐formed beneath the p‐type layer. The heterojunction of the two semiconductor materials is observed to be vertically constructed atop the parylene layer (Figure 3c). In the GA‐transistor (Figure 3d), when *V*_Screen‐G_ and *V*_CG_ are held constant, increasing *V*_DS_ from −10 to −30 and −50 V elevates the energy level and steepens the band slope, thereby enhancing the electric field across the channel. Because the carrier density (σ) in the p‐type channel remains fixed under identical gate bias conditions, the current density (J) scales proportionally with the electric field (E) in accordance with Ohm's law (J = σE). Figure 3e,f illustrates the Gaussian‐shaped transfer mechanism. Specifically, when *V*_CG_ is more negative than *V*_peak_ (Figure 3e), the p‐type channel experiences a stronger gate‐induced modulation but a reduced electric field, resulting in a flatter band slope, while the n‐type channel exhibits the opposite behavior. Consequently, as electrons become increasingly susceptible to drift and diffusion, the overall current level rises. Conversely, when *V*_CG_ is more positive than *V*_peak_ (Figure 3f), the p‐type channel develops a steeper slope under a stronger electric field, whereas the n‐type channel exhibits a flatter slope under weaker field influence. As a result, holes become more susceptible to drift and diffusion, leading to a decrease in the current level. Following the sigmoid function analysis, we next focus on the Gaussian‐shaped transfer characteristics and their key tunable parameters.

The Gaussian activation function has controllable parameters,^[^ ^60^, ^61^, ^62^ ^]^ including the mean (*µ*), amplitude (*A*), and standard deviation (*σ*) (Figure 3g):

(2) $$f\left(x\right)=A\cdot e^{-\frac{{\left(x-\mu \right)}^{2}}{2\sigma^{2}}}$$

where, *µ* determines the center of the Gaussian function and serves a role similar to the *V*_th_ in a transistor. When the voltage reaches *µ*, the current changes most significantly, marking the critical point where the channel is fully formed. *A* is the coefficient that sets the peak value of the Gaussian function, corresponding to *I*_peak_, the maximum current a transistor can achieve when fully turned on. The larger *A* value results in a higher maximum current, indicating greater carrier density and stronger current‐driving capability. *σ* determines the width of the Gaussian distribution and controls the rate of current change in the electrical characteristics of the GA‐transistor. The small *σ* value leads to a steep current transition, meaning sharp switching behavior. Conversely, a larger *σ* causes a more gradual current increase, enabling smooth switching operation. Modulating the characteristics of *µ*, *A*, and *σ* is a crucial task, as it can alter computing accuracy and computational speed. We extracted *µ*, *A*, and *σ* from the transfer curve of the GA‐transistor to verify the ability to control Gaussian activation function parameters by adjusting *V*_DS_ and *V*_Screen‐G_. Figure S18 (Supporting Information) presents the results of Gaussian parameter modulation with respect to *V*_DS_ (from −30 to −50 V) under a fixed *V*_Screen‐G_ of −10 V, whereas Figure 3h shows the effect of varying *V*_Screen‐G_ on the Gaussian characteristics. When *V*_DS_ increases from −30 to −50 V, *A* is increased from 0.09 to 1.05 µA, and µ is increased from 0.01 V to 5.85 V. The reason is that, as mentioned earlier, the charge injection increased, and the conductivity of both the n‐channel and p‐channel improved. *σ* exhibits its highest value (*σ* = 5.9 V at *V*_DS_ = −30 V) when *V*_DS_ is small, due to the fixed *V*_Screen‐G_ of −10 V relative to the electrode on top of PH‐BTBT‐10, which causes the current to follow a saturation‐like sigmoid curve. However, as *V*_DS_ becomes more negative, the saturation effect decreases, and the maximum current increases, resulting in a decreasing trend of *σ* (*σ* = 3.72 V at *V*_DS_ = −42 V). Once *V*_DS_ exceeds −42 V, the current increase and the negative shift of the peak point cause *σ* to increase again (*σ* = 4.06 V at *V*_DS_ = −50 V). On the other hand, when *V*_DS_ is fixed and *V*_Screen‐G_ is increased, the channel of PH‐BTBT‐10 is more strongly formed, leading to an increase in conductivity and a negative shift in *V*_peak_. As a result, *A, µ*, and *σ* all increased, with *A* increasing from 0.02 to 0.35 µA, µ shifting from −1.94 to −5.75 V, and *σ* increasing from 1.22 to 3.57 V. Additional results related to the cosine similarity are shown in Figure S19 (Supporting Information).

Additionally, device‐to‐device variability and deviations from ideal sigmoid and Gaussian functions were quantitatively analyzed (see Experimental Section for detailed modeling methodology and device non‐ideality). Using the advantages of organic materials, we also demonstrated flexible SA‐ and GA‐transistors fabricated on namecard substrates (Figure S20a, Supporting Information). Both devices maintained stable operation under 900 bending cycles at 25°, and their activation‐function shapes (sigmoid and Gaussian) were preserved with negligible distortion even at bending angles up to 45° (Figure S20b–e, Supporting Information). These results confirm the mechanical robustness of our transistors, underscoring their potential for flexible and wearable neuromorphic systems.

**Figure 4:**
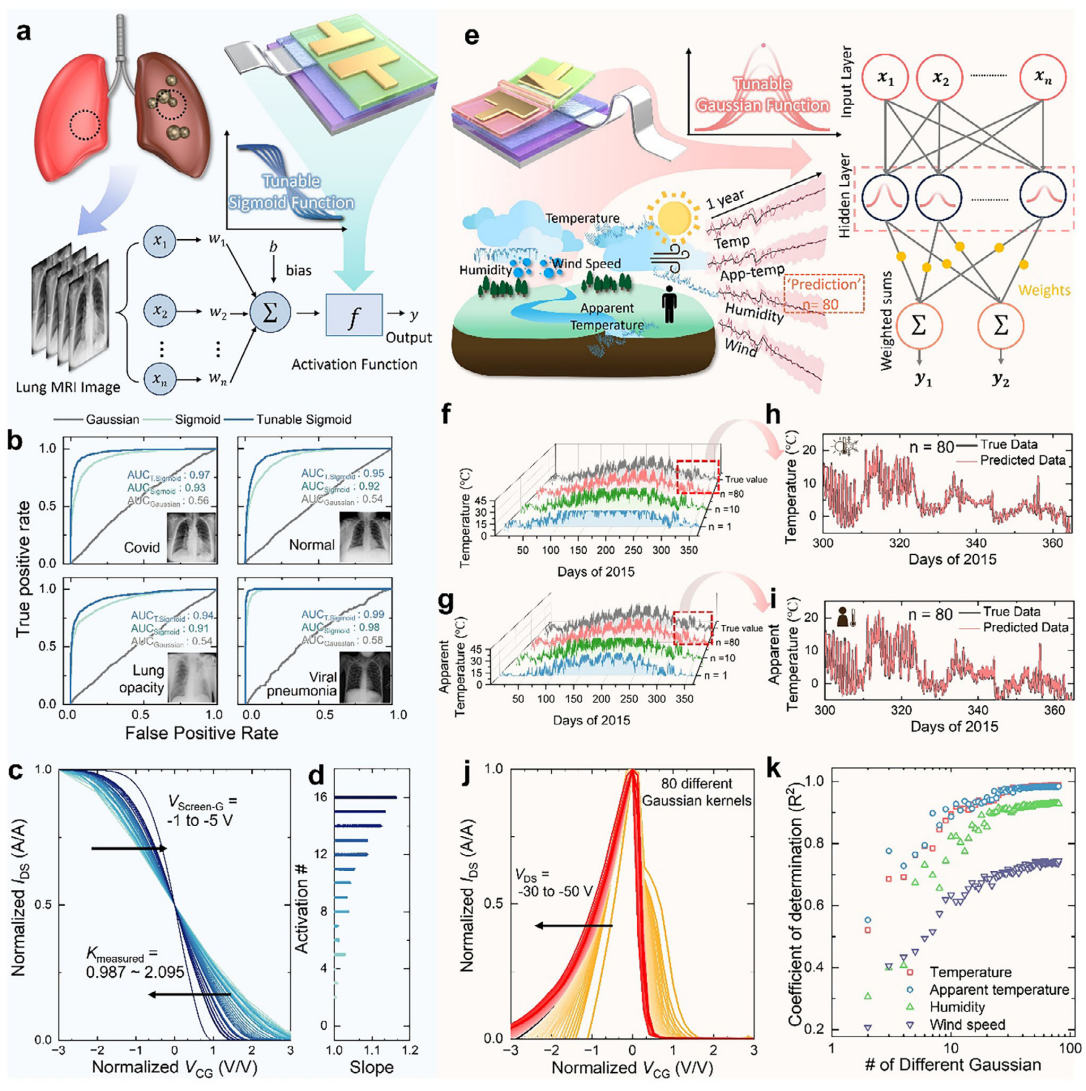
Application of a transistor‐level sigmoid activation function for lung MRI image classification. a) Schematic overview of the MRI‐based classification task with four clinically relevant categories: COVID, Normal, Lung opacity, and Viral Pneumonia. b) ROC curves showing true positive rate vs false positive rate, confirming improved diagnostic classification performance and higher AUC values achieved with tunable sigmoid activations with SA‐transistor. c) Normalized *I*_DS_ vs normalized *V*_CG_ characteristics of the SA‐transistor measured under different *V*_Screen‐G_, demonstrating that the slope of the sigmoid function can be precisely tuned using our device. d) Learned sigmoid slopes across different layers after training, showing adaptive slope tuning within the experimentally achievable range of the SA‐transistor. Application of a transistor‐level Gaussian activation function for time‐series environmental data prediction. e) Schematic diagram of a hardware‐based RBF network utilizing the GA‐transistor for analyzing environmental parameters including temperature, apparent temperature f,g). Predicted (colored lines) vs actual (black solid line) environmental measurements for (f) temperature, (g) apparent temperature showing enhanced predictive accuracy as the number of Gaussian kernels increases (blue: 1 kernel, green: 10 kernels, red: 80 kernels). h,i) Direct comparison between actual (*x*‐axis) and predicted (*y*‐axis) values for (h) temperature, (i) apparent temperature at the highest kernel count (*N* = 80), highlighting excellent prediction accuracy. j) *I*_DS_ vs *V*_CG_ characteristics of the GA‐transistor measured under different *V*_DS_, demonstrating precise tuning capability of the *σ* and confirming that our GA‐transistor can experimentally realize 80 distinct Gaussian kernels. k) *R* ^2^ vs the number of Gaussian kernels, quantitatively confirming the significant improvement in prediction accuracy with increased kernel count, emphasizing the practical advantages of the GA‐transistor's single‐device Gaussian implementation.

Neural network architectures critically rely on nonlinear activation functions, particularly sigmoid and Gaussian types, to capture complex data patterns efficiently.^[^ ^63^, ^64^ ^]^ Sigmoid functions, characterized by smooth, monotonic transitions, excel in deep networks by facilitating stable gradient propagation across multiple layers,^[^ ^65^ ^]^ whereas Gaussian‐based radial basis functions (RBFs) efficiently model temporally dynamic patterns typical in time‐series data.^[^ ^66^, ^67^ ^]^ However, practical hardware implementations of these activation functions have historically faced significant challenges,^[^ ^62^, ^68^ ^]^ often involving complicated analog circuits or extensive digital logic arrays. Such implementations typically require intricate transistor networks, operational amplifiers, and feedback loops, resulting in increased hardware complexity, power consumption, device footprint, and sensitivity to parameter drift.^[^ ^69^, ^70^ ^]^ By enabling tunable sigmoid and Gaussian responses within a single device, our approach directly supports practical applications such as medical image classification and forecasting applications. **Figure** **4**a shows a schematic overview of a lung MRI image classification task involving four clinically relevant categories: COVID, normal, lung opacity, and viral Pneumonia. Accurate classification of these images significantly impacts clinical diagnostics and decision‐making. To evaluate the sigmoid activation performance, we utilized a LiteResNet‐18 deep neural network architecture (Detailed network structure and training methodology are provided in the Experimental Section), as shown in Figure S21 (Supporting Information). The activation function is critical as it introduces the nonlinearity necessary for complex feature extraction. To examine the effect of the tunable sigmoid, the slope of the activation function was included as a trainable parameter, initialized to unity and updated during backpropagation together with other network weights. Figure S22 (Supporting Information) shows classification accuracy vs training epoch for networks employing fixed Gaussian, fixed sigmoid, and tunable sigmoid activations. Networks with Gaussian activations (red line) saturate quickly, limiting gradient propagation and causing early stagnation of accuracy (≈48%). In contrast, fixed sigmoid activation (light blue line) achieves continued accuracy improvement (≈77%) due to enhanced gradient flow in deeper layers. Crucially, the tunable sigmoid activation (dark blue line), where sigmoid slopes are adaptively learned through gradient backpropagation, achieves the highest accuracy (≈84%), demonstrating that dynamically optimizing the sigmoid slope significantly enhances network performance. Figure 4b shows the receiver operating characteristic (ROC) curves (true positive rate vs false positive rate), which illustrate the trade‐off between the true positive rate (TPR) and the false positive rate (FPR) as the decision threshold is varied. These quantities are defined as follows:^[^ ^71^ ^]^

(3) $$FPR=\frac{FP}{FP+TN}$$

(4) $$TPR=\frac{TP}{TP+FN}$$

where true positive (TP) denotes the number of positive samples correctly classified, and false negative (FN) refers to positive samples incorrectly classified as negative. Thus, TPR represents the proportion of actual positive cases that are correctly predicted, while FPR indicates the proportion of negative samples that are incorrectly predicted as positive. To quantify this performance, the area under the curve (AUC) is used, calculated using the trapezoidal rule:^[^ ^72^ ^]^

(5) $$\mathrm{AUC}=\sum_{\left(i=1\right)}^{\left(n-1\right)}\left(x_{\left(i+1\right)}-x_{i}\right)\cdot \frac{y_{i+1}+y_{i}}{2}$$

where and are the FPR and TPR values at the i‐th threshold, respectively. An AUC of 1.0 indicates a perfect classifier, 0.5 corresponds to a random guess, and values below 0.5 suggest inverse prediction performance. These results confirming that tunable sigmoid activation consistently yields superior AUC values compared to fixed sigmoid and Gaussian across all diagnostic categories.

The confusion matrix of the model trained with the tunable sigmoid activation is presented in Figure S23 (Supporting Information). Figure 4c shows normalized *I*_DS_ vs normalized *V*_CG_ characteristics of the SA‐transistor measured under different *V*_Screen‐G_, demonstrating that the slope of the sigmoid function can be precisely tuned using our device. Figure 4d shows learned sigmoid slopes vs layer number after training completion, illustrating adaptive slope tuning. Initially set at unity, slopes evolved adaptively between ≈1.0 to 1.2, a range fully accommodated by our SA‐transistor tuning capability (0.9875–2.095). This adaptive tuning confirms the compatibility and feasibility of practical hardware implementation using the SA‐transistor.

While sigmoid activations excel in complex image classification tasks, Gaussian activation functions uniquely benefit analyses of simpler yet temporally structured datasets. Figure 4e shows a schematic of the hardware‐based RBF network, employing our GA‐transistor to analyze environmental time‐series data (temperature, apparent temperature, humidity, and wind speed). Historical data (2010–2014) served as training, with 2015 as an independent test set (see Experimental Section for detailed network architecture and training methods). Figure 4f,g and Figure S24a,b (Supporting Information) show predicted vs actual measurements for temperature (Figure 4f), apparent temperature (Figure 4g), humidity (Figure S24a, Supporting Information), and wind speed (Figure S24b, Supporting Information). Actual data (black solid lines) and predictions generated by varying numbers of Gaussian kernels (red: 3 Gaussians, green: 10 Gaussians, blue: 80 Gaussians) clearly demonstrate enhanced accuracy as the number of Gaussian kernels increases. Figure 4f,i compare predicted and actual data for temperature (Figure 4h), apparent temperature (Figure 4i), humidity (Figure S24c, Supporting Information), and wind speed (Figure S24d, Supporting Information), respectively, at *N* = 80. These plots highlight the excellent predictive accuracy achieved by the RBF model configured with 80 Gaussian kernels. To ensure consistency between simulation and experimental results, Gaussian kernels were directly derived from experimentally measured *σ* values, as shown in Figure 4j. Specifically, the 80‐kernel configuration (*N* = 80) employed all 80 measured *σ* values, while smaller kernel numbers (e.g., *N* = 3 or 10) utilized *σ* values selected at equal intervals from this experimentally obtained set (3 Gaussians: 1st, 41st, 80th; 10 Gaussians: 1st, 11th, 21st, …, 80th), ensuring realistic and hardware‐consistent conditions. Figure 4k shows the coefficient of determination (*R* ^2^) vs the number of Gaussian kernels, quantitatively confirming that increasing the number of Gaussian kernels significantly reduces mean squared error (MSE). The MSE and mean absolute error (MAE) as functions of the number of Gaussian kernels are presented in Figure S25 (Supporting Information). The effect of non‐ideal Gaussian distortion on RBF network accuracy was evaluated in our simulations, confirming that the measured level of deviation results in only modest performance degradation (Figure S26, Supporting Information). This highlights the practical importance and substantial advantage of our single‐device Gaussian implementation for efficient hardware‐based neuromorphic computing. It should be noted that when integrating the proposed SA‐ and GA‐transistors into practical neural network hardware, both the input gate voltage and output drain current must be normalized to the [0–1] range typically required by accelerators. This normalization can be readily achieved using compact, well‐established peripheral circuits such as a voltage shifter–scaler for input mapping and a Gilbert current‐mode normalizer for output scaling. Because such analog interface blocks are already standard practice in neuromorphic systems, they impose negligible overhead in area or power while ensuring compatibility with conventional architectures. Therefore, normalization does not represent a unique limitation of our approach but rather a routine design step naturally accompanying transistor‐level activation functions.

**Figure 5:**
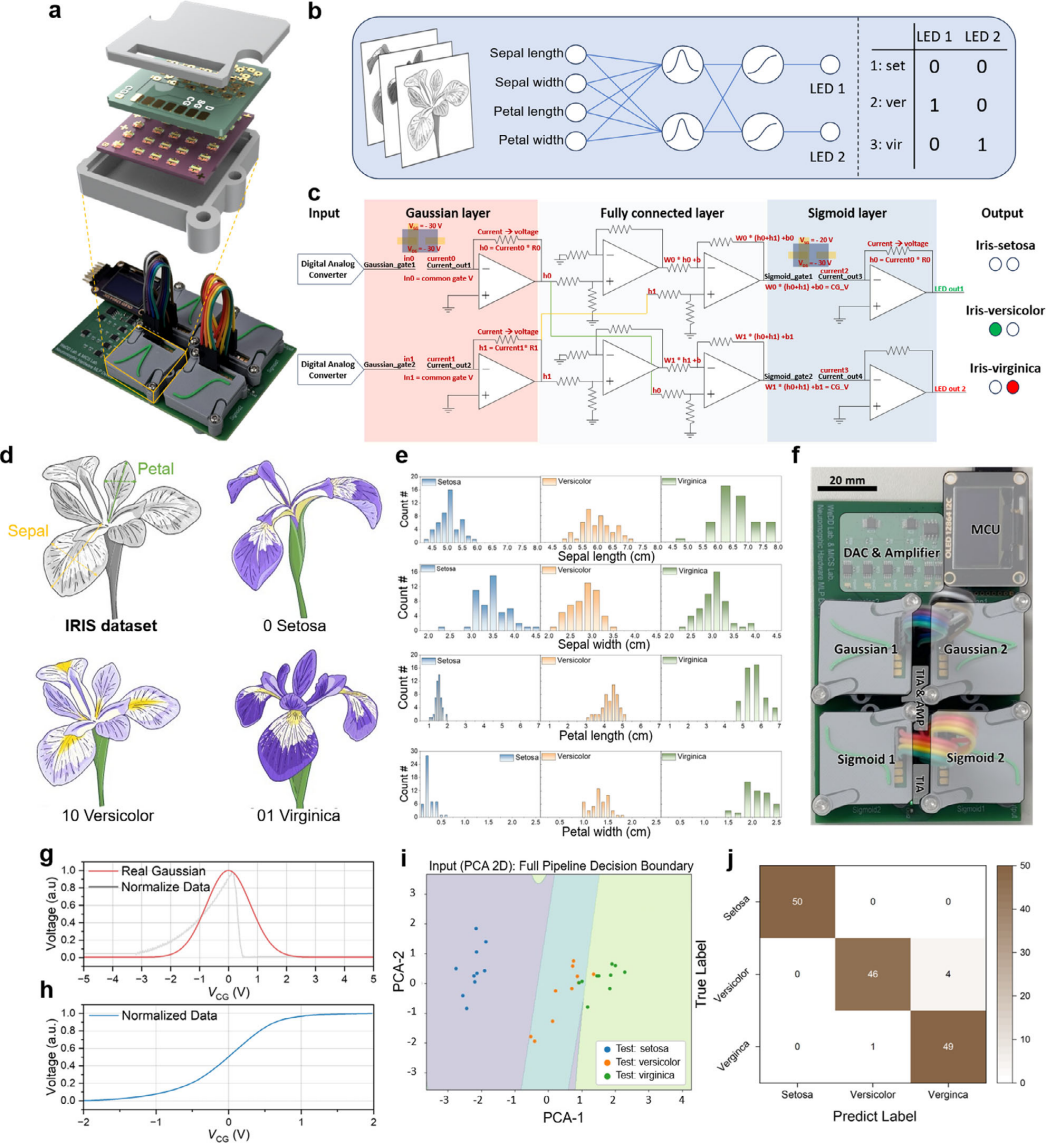
a) Image and Schematic of a fully hardware‐based MLP demonstration system. b) Illustration of the iris classification MLP structure. c) Circuit iris classification MLP system using SA‐transistor and GA‐transistor. d) The characteristics of the IRIS dataset and the biological basis for classification. e) Distribution of the four input features across the three classes (setosa, versicolor, virginica). f) PCB integrating DAC, amplifier, and MCU with sockets for two SA‐transistors and two GA‐transistors. The g) Gaussian and h) sigmoid activation behavior of the proposed devices. i) Decision boundary and j) Confusion matrix of the hardware‐implemented MLP classification system visualized in 2D PCA space.

To validate the system‐level applicability of the proposed activation‐function transistors, we implemented a fully hardware‐based multilayer perceptron (MLP) demonstration system. **Figure** **5**a illustrates the integrated hardware setup, including the assembled printed circuit board (PCB) and socket jig designed for direct connection with our transistor arrays. The overall MLP structure used in the demonstration is shown in Figure 5b. The model was trained to classify data from the IRIS dataset using four input features (sepal length, sepal width, petal length, and petal width). These features were weighted and summed into two hidden‐layer neurons employing Gaussian‐like activation functions, followed by two output‐layer neurons using sigmoid‐like activation functions. The final output was encoded into binary (00 = setosa, 01 = versicolor, 10 = virginica), allowing direct light‐emitting diode (LED)‐based visualization without analog‐to‐digital conversion.

The detailed hardware implementation of this MLP structure is shown in Figure 5c. Analog input voltages generated by a digital‐to‐analog converter (DAC) were applied to the gate terminals of the Gaussian‐layer transistors. The resulting Gaussian‐shaped current responses were converted into voltage using transimpedance amplifiers (TIAs), then processed by weighted summing/differential amplifiers. These signals were subsequently fed into the gate terminals of the sigmoid‐like transistors, producing binary output currents that switched LEDs ON or OFF depending on the classification result. The characteristics of the IRIS dataset and the biological basis for classification are shown in Figure 5d, while the distribution of the four input features across the three classes is visualized in Figure 5e. The actual PCB consists of two Gaussian and two sigmoid transistor sockets, along with a microcontroller unit (MCU), DACs, TIAs, and analog amplifiers (Figure 5f). The activation behavior of the proposed devices prior to deployment is shown in Figure 5g,h. For the Gaussian function, normalized experimental data were fitted to a real Gaussian model. In contrast, the sigmoid function was applied using a simple normalization process. Normalization in the hardware‐based MLP was inherently achieved through the operation of the TIAs and subsequent analog amplifiers, which converted the raw *I*_DS_ of the SA‐ and GA‐transistors into voltage signals and adjusted their amplitude to fall within the normalized range of 0–1. This circuit‐level normalization ensured that the device outputs were directly compatible with the standardized activation domain required for MLP processing.

Figure 5i shows the decision boundary of the hardware‐implemented MLP classification system visualized in 2D principal component analysis (PCA) space. The four input features from the IRIS dataset (sepal length, sepal width, petal length, petal width) were first projected onto two principal components (PCA‐1 and PCA‐2) for visualization. The decision regions for the three output classes (setosa, versicolor, and virginica) are clearly separated, and the test data points (colored by true labels) are mostly located within their correct classification regions. This confirms that the Gaussian‐like and sigmoid‐like activation transistors effectively emulate nonlinear mappings even under analog operation. To quantitatively evaluate classification performance, a confusion matrix is presented in Figure 5j. The hardware system achieved 100% accuracy for the setosa class, while the versicolor and virginica classes were classified with 92% and 98% accuracy, respectively. Out of 150 test samples, only 5 were misclassified, resulting in an overall accuracy of 96.7%. These results validate that the proposed analog MLP system can perform robust and reliable inference on real‐world datasets without the need for digital post‐processing or analog‐to‐digital converter (ADC) conversion. To validate our approach, we provide a supporting video demonstration of a real‐time hardware implementation of MLP. These device‐level activation functions are directly compatible with neuromorphic accelerators, where SA‐ and GA‐transistors can serve as compact, tunable activation modules following analog dot‐product engines or matrix–vector multipliers.^[^ ^73^, ^74^, ^75^ ^]^

## 3 Conclusion

The adoption of asymmetric screen gates allows for precise modulation of the electrical characteristics in the SA‐transistor and GA‐transistor. Key parameters of the activation functions (i.e., threshold position, slope, saturation current, mean, amplitude, and standard deviation) can be finely controlled through gate biasing. These device‐level activation functions address the inherent limitations of conventional circuit‐based or software‐based implementations by significantly reducing system complexity, footprint, and power consumption. Furthermore, the tunability of activation responses at the device level enhances computational accuracy and processing efficiency. The SA‐transistor demonstrated its applicability in deep learning tasks, such as lung MRI classification, by improving prediction accuracy through adaptive slope control. The GA‐transistor achieved efficient time‐series forecasting through hardware‐compatible Gaussian kernel scaling, delivering high predictive performance with minimal circuit overhead. By integrating screen gate into a SA‐transistor and a GA‐transistor, tunable activation functions can be implemented, underscoring the potential of this approach for analog device‐based AI computing hardware. In addition, the fully hardware‐based MLP system built from these devices achieved 96.7% accuracy on the IRIS dataset, validating their system‐level applicability. This demonstration highlights that screen gate‐based SA‐ and GA‐transistors not only function as compact, tunable modules at the device level but also enable robust, low‐power, and ADC‐free inference when integrated into larger neuromorphic architectures.

## 4 Experimental Section

### Fabrication and Characterization of SA‐transistor and GA‐transistor

The SA‐transistors and GA‐transistors were fabricated using a bottom‐gate, top‐contact structure. A p‐doped silicon wafer with a 300 nm‐thick thermally grown SiO_2_ layer was used as the substrate, where the p‐type Si served as the common gate and the SiO_2_ as the gate dielectric. The substrates were cleaned by sequential ultrasonic treatment in acetone and isopropyl alcohol for 15 min each, rinsed with deionized water, and dried with high‐purity nitrogen. A 70 nm‐thick aluminum layer was deposited by thermal evaporation (at a rate of ≈10 Å s^−1^) to form the screen gate, followed by deposition of a 300 nm‐thick parylene layer using a parylene coater as an insulating layer. For the SA‐transistor, a ≈30 nm‐thick PH‐BTBT‐10 layer was deposited by thermal evaporation at a rate of ≈0.3 Å s^−1^ and annealed at 80 °C for 30 min. For the GA‐transistor, the same PH‐BTBT‐10 deposition and annealing were carried out, followed by thermal evaporation of a ≈30 nm‐thick PTCDI‐C13 layer at a rate of ≈0.3 Å s^−1^ to form a heterojunction channel. A 70 nm‐thick Au source and drain electrodes were deposited by thermal evaporation, where the first ≈10 nm was grown at ≈0.3 Å s^−1^ and the remaining thickness up to ≈70 nm was grown at ≈1 Å s^−1^. The device dimensions were fixed at 2600 µm in width and 1000 µm in length. For the electrical characteristic to measure current–voltage (*I*–*V*) behavior, the probe station (Keithley 4200A) was used.

### Low‐Frequency Noise Analysis of GA‐ and SA‐Transistors

To provide physical justification beyond curve fitting, low‐frequency noise was measured, and the normalized power spectral density (*S*_ID_/*I*_DS_^2^) of both the GA‐ and SA‐transistors were analyzed under various gate biases. Representative transfer curves are shown in Figure S27 (Supporting Information). In Figure S28 (Supporting Information), *S*_ID_/*I*_DS_^2^ (sampled at 100 Hz) was plotted vs *V*_CG_. For the GA‐transistor (Figure S28a, Supporting Information), the noise decreases as *V*_CG_ is swept from –8 to –1 V, and then increases again from +1 to +8 V. This non‐monotonic trend reflects the anti‐ambipolar series stack: at negative *V*_CG_ only the p‐type branch conducts, so higher carrier density suppresses relative fluctuations; at positive *V*_CG_ both p‐ and n‐type branches conduct, activating more traps and scatterers and thus enhancing relative fluctuations. By contrast, the SA‐transistor (Figure S28b, Supporting Information) exhibits a monotonic decrease of *S*_ID_/*I*_DS_^2^ from –2 to –10 V as carriers accumulate in the single p‐type channel. Beyond –10 V, the curves saturate (–10 to –16 V) since additional gate bias no longer significantly changes the carrier population, consistent with the flat top of the sigmoid‐like transfer characteristics. Figures S29 and S30 (Supporting Information) show *S*_ID_/*I*_DS_^2^ vs frequency at representative gate biases. All spectra exhibit clear 1/*f* behavior with similar slopes in log–log plots. For the GA‐transistor, the lowest noise floor appeared near *V*_CG_ = –1 V, rising again at positive *V*_CG_ when both branches conducted. For the SA‐transistor, the 1/*f* magnitude steadily decreased with more negative *V*_CG_ until full channel accumulation, beyond which spectra collapsed onto each other, evidencing the saturation regime. These bias‐dependent noise signatures corroborated the transport mechanisms responsible for the sigmoid‐like and Gaussian‐like *I*–*V* characteristics observed in Figures 1 and 2 of the main text.

### Modeling Methodology and Device Non‐Ideality

To assess how measured device characteristics influence the simulations, activation‐function parameters were extracted directly from experimental transfer curves of 50 individual SA‐ and GA‐transistors. Figures S31 and S32 (Supporting Information) present the distributions of MSE and *R* ^2^ relative to ideal sigmoid and Gaussian functions, respectively. The extracted results indicated that SA‐transistors exhibited close agreement with a sigmoid‐like activation function, while GA‐transistors displayed measurable but manageable deviations within the tested range. Figures S33 and S34 (Supporting Information) illustrate representative transfer curves with error bars across devices, confirming device‐to‐device variability. These analyses indicated that the modeling incorporates realistic device parameters and accounts for non‐idealities observed in the measurements.

In the RBF simulation, the *N* = 80 setting used all measured σ values; for smaller *N*, they were subsample them at equal intervals so the kernels remain hardware‐consistent. To mimic measured distortion, perturbed widths σ′ were formed by applying bounded multiplicative noise, σ′_k_ = clamp(σ_k_·(1+ε_k_)), where ε_k_ is drawn from a zero‐mean distribution (or a smooth sinusoidal pattern across k) and the clamp keeps σ′ within the empirical [min, max] of the measurements. For a sweep of distortion levels, predictions were recomputed and two quantities were logged: a kernel‐space error (mean_k_(σ′_k_−σ_k_)^2^) and the prediction mean squared error (MSE) on the test set, then plot them against each other. The sweep range was anchored to the device‐level variability, it is quantified from 50 transistors (MSE/*R* ^2^ vs ideal activations), and included the measured Gaussian distortion level (≈1.4 × 10^−3^ MSE). Consistent with the manuscript, this procedure showed that distortions at the measured magnitude cause only modest accuracy degradation, i.e., the architecture was tolerant to realistic device imperfections.

### Sigmoid‐Based LiteResNet‐18 Network Simulation Details

To systematically evaluate the effectiveness of transistor‐level sigmoid activation functions, image classification tasks were conducted using lung MRI images classified into four diagnostic categories: COVID, Normal, Lung opacity, and Viral Pneumonia. The dataset was divided into training (80%) and test (20%) sets for performance evaluation. Data augmentation techniques, including random horizontal flipping (probability 0.5) and random rotation (±15°), were applied exclusively to the training set to enhance generalization. All images were resized to 224 × 224 pixels and normalized using ImageNet‐derived mean and standard deviation values.

The LiteResNet‐18 architecture employed in this study is a compact variant of the standard ResNet‐18, specifically tailored for reduced computational complexity. The network comprises four convolutional blocks with progressively increasing channel dimensions: 16, 32, 64, and 128, respectively. Each convolutional block consists of two convolutional layers followed by batch normalization and an activation function. To systematically compare the effects of different activation strategies, three scenarios were implemented:

- Fixed Sigmoid: Utilized a conventional sigmoid activation function without learnable parameters.
- Fixed Gaussian: Implemented a standard Gaussian activation with fixed parameters (*µ* = 0, *σ* = 1).
- Tunable Sigmoid: Employed a sigmoid activation function with learnable slope parameters, optimized via gradient backpropagation during training. Initially, all slope parameters were set to unity (1.0).

The network parameters were initialized using Xavier normal initialization for convolutional and fully connected layers, while batch normalization layers were initialized with weights of 1 and biases of 0. Training proceeded for a total of 100 epochs using the Adam optimizer with a base learning rate of 3 × 10^−6^ and weight decay of 1 × 10^−6^. For the tunable sigmoid network, activation parameters (a and b) were trained with a separate, slightly higher learning rate (1 × 10^−5^) to facilitate efficient parameter convergence. Learning rates were updated each epoch via a step‐wise decay scheduler with a gamma factor of 0.99. Performance metrics (training and test accuracy, precision, recall, and confusion matrices) were evaluated at every epoch to thoroughly assess classification accuracy and model stability. Additionally, the evolution of the sigmoid activation slope parameter was continuously tracked across each network layer throughout the entire training process. True positive rates (TPR), false positive rates (FPR), and areas under the curve (AUC) were systematically documented for comprehensive analysis. Note that all simulations were performed using PyTorch on an NVIDIA GPU with CUDA support.

### Gaussian‐Based RBF Network Simulation Details

To systematically evaluate the efficacy of hardware‐level Gaussian activation functions, a radial basis function (RBF) neural network was constructed, specifically designed for analyzing time‐series data. Environmental datasets (comprising temperature, apparent temperature, humidity, and wind speed recorded hourly) were utilized to demonstrate the model's capabilities. Raw data were sourced from a publicly available weather dataset, which underwent preprocessing steps involving conversion of timestamps from string format to UTC datetime, removal of timezone information, and chronological sorting. Data from 2010 to 2014 were designated as the training set, while data from the year 2015 served as an independent test set. The RBF network employed a sliding‐window approach (look_back = 10), wherein each input vector consisted of ten consecutive hourly data points to predict the subsequent hour's value. Prior to training, both input and target data underwent standardization (zero mean, unit variance) to ensure numerical stability and facilitate effective model convergence. Gaussian kernels within the network were defined by two critical parameters: the center (mean) and standard deviation (*σ*). Kernel centers were determined using the K‐means clustering algorithm applied directly to the training dataset, ensuring representative and efficient coverage of the input space. A total of 200 Gaussian kernels were employed consistently across experiments. To faithfully represent hardware capabilities, experimentally measured σ values (80 distinct *σ* values ranging from 0.5 to 1.45, as shown in Figure 4j) were directly employed in simulation. For configurations utilizing fewer than 80 Gaussian kernels (*N* <80), σ values were selected at equal intervals from the sorted list of measured 80 *σ* values. For instance, for *N* = 3, the 1st, 41st, and 80th σ values were used, whereas for *N* = 10, *σ* values at intervals of every 9 positions (1st, 10th, 19th, etc.) were selected. This systematic extraction ensured consistent coverage of the experimentally accessible σ range, accurately reflecting the hardware's tuning capability. Model training involved calculating the activation responses through Gaussian functions based on the Euclidean distances between input vectors and the kernel centers identified via clustering. The final weights connecting the Gaussian hidden units to the output node were determined using linear least squares optimization. Model performance was quantitatively assessed using MSE, MAE, and *R* ^2^ scores computed on the independent test dataset. Note that all simulations were performed using Python with the libraries NumPy, pandas, scikit‐learn, and matplotlib.

## Conflict of Interest

The authors declare no conflict of interest.

## Supporting information



Supporting Information

Supporting Information

Supporting Information

## Data Availability

The data that support the findings of this study are available from the corresponding author upon reasonable request.
