# Supplementary material for: Transistor‐Level Activation Functions via Two‐Gate Designs: From Analog Sigmoid and Gaussian Control to Real‐Time Hardware Demonstrations
Source: Adv Mater. 2025 Nov 24;38(21):e11018. doi: 10.1002/adma.202511018 (PMC13073066; doi:10.1002/adma.202511018)
Supplement: Supplementary file 1 — Supporting Information [file ADMA-38-e11018-s001.docx]

Supporting Information

Transistor-Level Activation Functions via Two-Gate Designs: From Analog Sigmoid and Gaussian Control to Real-Time Hardware Demonstrations

Junhyung Cho^1‡^, Youngmin Han^2‡^, Won Woo Lee^1‡^, Youngwoo Yoo^3‡^, Kannan Udaya Mohanan^4^, Chang-Hyun Kim^4^, Junhwan Choi^5^, Young-Joon Kim^3,*^, Wonjun Shin^6,*^ and Hocheon Yoo^2,*^

^1^Department of Artificial Intelligence Semiconductor Engineering, Hanyang University, 222 Wangsimni-ro, Seoul 04763, Republic of Korea

^2^Department of Electronic Engineering, Hanyang University, 222 Wangsimni-ro, Seoul 04763, Republic of Korea

^3^Department of Semiconductor Engineering, Gachon University, Seongnam-si, Gyeonggi-do, 13120, Republic of Korea

^4^School of Electrical Engineering and Computer Science, University of Ottawa, Ottawa, ON K1N 6N5, Canada

^5^Department of Chemical Engineering, Dankook University, 152 Jukjeon-ro, Suji-gu, Yongin, Gyeonggi-do 16890, Republic of Korea

^6^Department of Semiconductor Convergence Engineering, Sungkyunkwan University, Suwon, 16419, Republic of Korea

^‡^ J. Cho, Y. Han, W. W. Lee, Y. Yoo contributed equally to this work.

E-mail: youngkim@gachon.ac.kr (Y. Kim), swj0107@skku.edu (W. Shin), hocheon@hanyang.ac.kr (H. Yoo)

Keywords: screen gate structure, sigmoid activation function, Gaussian activation function, anti-ambipolar transistor, prediction of change; multilayer perceptron; hardware application;

**<Table of Contents>**

**Supporting Figures**

**Figure S1.** OM images of (a) the corresponding SA-transistor and its device dimensions, (b) the fabrication sequence for the sigmoid-like activation transistor; (i) screen gate evaporated through shadow mask; (ii) parylene dielectric evaporated full-surface; (iii) PH-BTBT-10 evaporated through shadow mask and annealed at 80℃; (iv) Au electrode evaporated through shadow mask. (c) the corresponding GA-transistor and its device dimensions, and (d) the fabrication sequence for the Gaussian-like activation transistor; (i) screen gate evaporated through shadow mask; (ii) parylene dielectric evaporated full-surface; (iii) PH-BTBT-10 evaporated through shadow mask and annealed at 80℃; (iv) PTCDI-C13 evaporated through shadow mask; (vi) Au electrode evaporated through shadow mask.

**Figure S2.** OM image of the AFM-measured region (a) and the screen-gate structure (b), and AFM step-height profiles (c) at [1] electrode/PH-BTBT-10, [2] PH-BTBT-10/heterojunction, [3] screen gate/heterojunction, [4] heterojunction/PTCDI-C13, and [5] PTCDI-C13/electrode. [6] parylene/screen gate.

**Figure S3.** (a) Cross-sectional SEM image of the SiO_2_/screen gate/parylene layers measured to confirm the proper deposition of the screen gate between the dielectric layers. (b) EDS mapping image of screen gate structure cross-section view.

**Figure S4.** Surface SEM images of the GA-Transistor heterojunction. SEM images highlighting key material boundaries in the device: (a) The Au/PH-BTBT-10 to PH-BTBT-10 interface; (b) The PH-BTBT-10 to PTCDI-C13/PH-BTBT-10 heterojunction interface; (c) The PTCDI-C13/PH-BTBT-10 heterojunction to PTCDI-C13 interface.

**Figure S5.** EDS mapping images providing compositional analysis of the device structures: (a) EDS mapping of the SA-transistor and (b) EDS mapping of the GA-transistor.

**Figure S6.** Transfer characteristics of (a) a conventional three-terminal transistor and (b) a screen gate-inserted SA-transistor.

**Figure S7.** Transfer characteristics of the device with fixed *V*_DS_ of (a) −5 V and (b) −10 V. The *V*_Screen-G_ was varied from −1 V to −5 V with a step voltage of −0.1 V. The *V*_CG_ was swept from 20 V to −50 V.

**Figure S8.** Sigmoid fitting results of the transfer characteristics extracted at intervals of −0.5 V within the *V*_Screen-G_ range from −1 V to −5 V: (a) at *V*_DS_ = −5 V, and (b) at *V*_DS_ = −10 V. Solid lines denote the measured data, and dashed lines represent the fitted sigmoid functions.

**Figure S9.** Transfer characteristics measured with *V*_DS_ varied from −6 V to −10 V in steps of −0.1 V. The *V*_Screen-G_ was fixed at (a) −5 V and (b) −10 V for each measurement. The *V*_CG_ was swept from 20 V to −50 V.

**Figure S10.** Sigmoid fitting results of the transfer characteristics extracted at intervals of −0.5 V within the *V*_DS_ range from −6 V to −10 V: (a) at *V*_Screen-G_ = −5 V, (b) at *V*_Screen-G_ = −7.5 V, and (c) at *V*_Screen-G_ = −10 V. Solid lines represent the measured data, while dashed lines indicate the fitted sigmoid curves.

**Figure S11.** Cosine similarity between the measured transfer curves and the fitted sigmoid functions as a function of the *V*_DS_ varied from −6 V to −10 V.

**Figure S12.** Extracted values of the sigmoid parameters (a) *L*, (b) *k*, and (c) *X*_0_ as a function of the *V*_DS_ varied from −6 V to −10 V.

**Figure S13.** Extracted values of the sigmoid parameters (a) *L*, (b) *k*, and (c) *X*_0_ as a function of the *V*_Screen-G_ varied from −1 V to −5 V, when *V*_DS_ was −5, −7.5, and −10 V, respectively.

**Figure S14.** Extracted values of the sigmoid parameters (a) *L*, (b) *k*, and (c) *X*_0_ as a function of the *V*_DS_ varied from −6 V to −10 V, when *V*_Screen-G_ was −5, −7.5, and −10 V, respectively.

**Figure S15.** Drift-diffusion simulation on the fundamental mechanism of the SA-transistor. (a) Sigmoid-like transfer curve and its *V*_Screen-G_ control. (b) Comparison between the devices with and without the screen gate. (c) 2D hole distribution in the no-screen gate device. (d) 2D hole distribution in the sigmoid-like device (S: source, D: Drain).

**Figure S16.** 1D potential profiles that match the 2D hole distribution maps. These profiles were extracted 1 nm above the PH-BTBT-10/parylene interface to capture the region of carrier accumulation and surface charge transport. The vertical alignment with the source (S), drain (D), and screen gate (SG) electrodes in each device is shown as a visual guide.

**Figure S17.** Drift-diffusion simulation on the structural aspect of the SA-transistor. (a, b) Illustration and results on the effects of the position of the screen gate. (c, d) Illustration and results on the effects of the screen gate *L*_ext_ (S: source, D: drain, CG: common gate, SG: screen gate).

**Figure S18.** Extracted values of the Gaussian parameters (a) *A*, (b) *µ*, and (c) *σ* as a function of the *V*_DS_ varied from −30 V to −50 V.

**Figure S19.** Cosine similarity between the measured transfer curves and the fitted Gaussian functions as a function of (a) the *V*_DS_ varied from −30 V to −50 V, and (b) the *V*_Screen-G_ varied from −8 V to −10 V.

**Figure S20.** (a) The real image of SA-transistors and GA-transistors fabricated on a name card substrate. (b) Stability of key sigmoid parameters (*K*, *X*_0_, *L*) of the SA-transistor and (c) GA-transistor under 900 bending cycles (θ = 25°). (d) Transfer characteristics of the SA-transistor and (e) GA-transistor under different angles (θ = 0°, 25°, 45°).

**Figure S21.** Architecture of the LiteResNet-18 neural network used for evaluating various activation functions. Comparison of classification accuracy across training epochs for networks using fixed Gaussian, fixed sigmoid, and tunable sigmoid activations. The tunable sigmoid activation demonstrates superior performance, achieving approximately 84% accuracy.

**Figure S22.** Comparison of classification accuracy across training epochs for networks using fixed Gaussian, fixed sigmoid, and tunable sigmoid activations. The tunable sigmoid activation demonstrates superior performance, achieving approximately 84% accuracy.

**Figure S23.** Confusion matrix analysis of MRI image classification. Confusion matrix illustrating classification performance of the LiteResNet-18 neural network with tunable sigmoid activation function for lung MRI image classification.

**Figure S24.** (a, b) Predicted (colored lines) versus actual (black solid line) environmental measurements for (a) humidity, and (b) wind speed, showing enhanced predictive accuracy as the number of Gaussian kernels increases (blue: 1 kernel, green: 10 kernels, red: 80 kernels). (c, d) Direct comparison between actual (x-axis) and predicted (y-axis) values for (c) humidity, and (d) wind speed at the highest kernel count (*N*=80), highlighting excellent prediction accuracy.

**Figure S25.** Evaluation of prediction accuracy as a function of Gaussian kernels. (a) MSE versus the number of Gaussian kernels (b) MAE versus the number of Gaussian kernels.

**Figure S26.** Analysis of how the distortion (MSE) in Gaussian kernels, reflecting non-ideal device characteristics, affects the prediction accuracy (MSE of predicted signals) in the RBF network simulations.

**Figure S27.** (a) Transfer curve of the GA-transistor. (b) Transfer curve of the SA-transistor.

**Figure S28.** (a) *S*_ID_/*I*_D_² versus *V*_CG_ of the GA-transistor. (b) *S*_ID_/*I*_D_² versus *V*_CG_ of the SA-transistor.

**Figure S29.** (a) *S*_ID_/*I*_D_^2^ versus frequency of the GA-transistor at *V*_CG_ = −8 to −1 V. (b) *S*_ID_/*I*_D_^2^ versus frequency of the GA-transistor at *V*_CG_ = +1 to +8 V.

**Figure S30.** (a) *S*_ID_/*I*_D_² versus frequency of the SA-transistor at *V*_CG_ = −2 to −10 V. (b) *S*_ID_/*I*_D_² versus frequency of the SA-transistor at *V*_CG_ = −10 to −16 V.

**Figure S31.** Measured non-ideality across 50 Gaussian activation transistor devices expressed as (a) MSE and (b) *R*^2^.

**Figure S32.** Measured non-ideality across 50 sigmoid activation transistor devices expressed as (a) MSE and (b) *R*^2^.

**Figure S33.** Transfer curves (*I*_DS_ versus *V*_CG_) for 50 measured sigmoid activation devices, showing the mean curve and error bars representing the standard deviation across devices.

**Figure S34.** Transfer curves (*I*_DS_ versus *V*_CG_) for 50 measured Gaussian activation devices, showing the mean curve and error bars representing the standard deviation across devices.

**
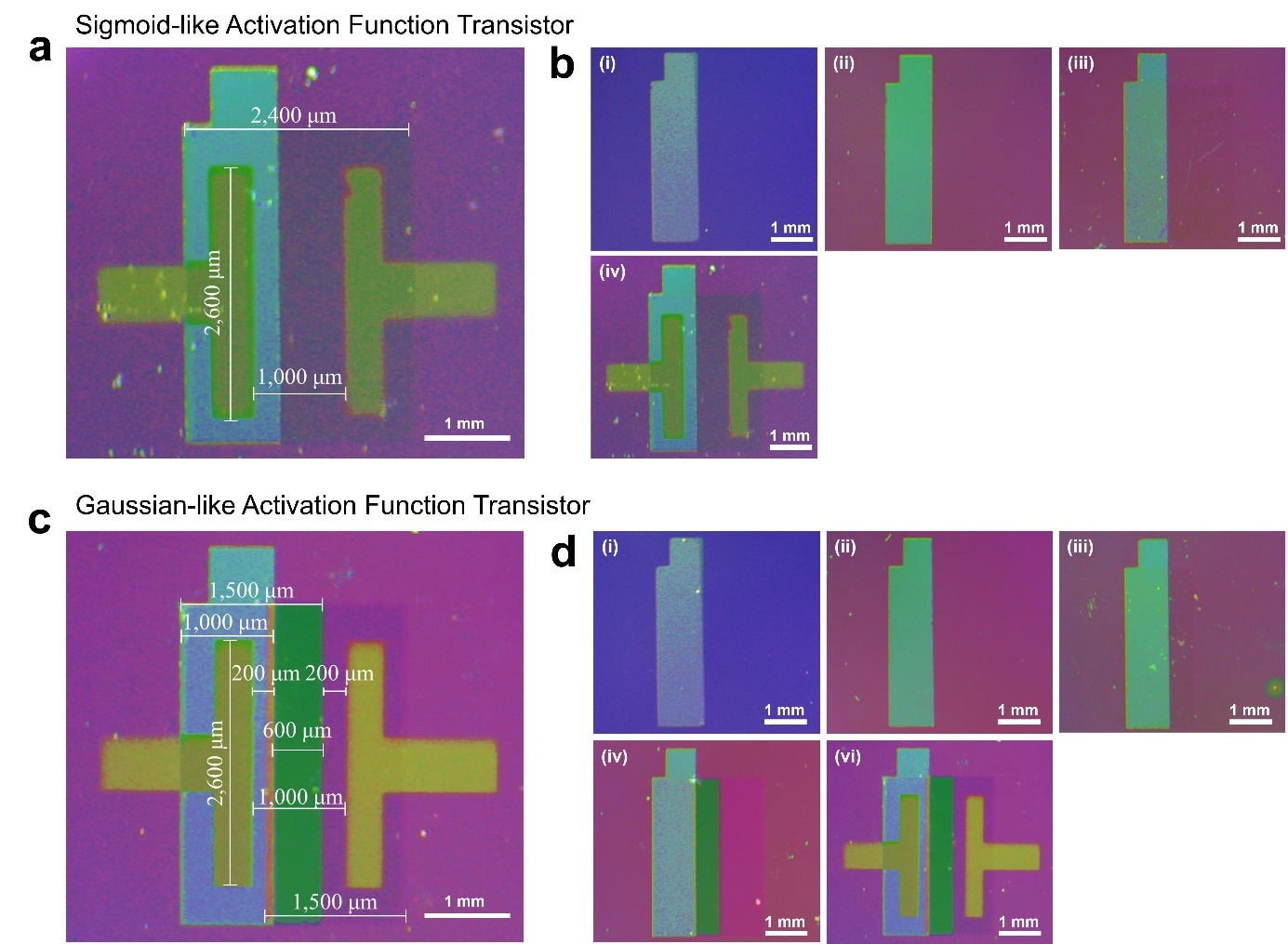
**

**Figure S1.** OM images of (a) the corresponding SA-transistor and its device dimensions, (b) the fabrication sequence for the sigmoid-like activation transistor; (i) screen gate evaporated through shadow mask; (ii) parylene dielectric evaporated full-surface; (iii) PH-BTBT-10 evaporated through shadow mask and annealed at 80℃; (iv) Au electrode evaporated through shadow mask. (c) the corresponding GA-transistor and its device dimensions, and (d) the fabrication sequence for the Gaussian-like activation transistor; (i) screen gate evaporated through shadow mask; (ii) parylene dielectric evaporated full-surface; (iii) PH-BTBT-10 evaporated through shadow mask and annealed at 80℃; (iv) PTCDI-C13 evaporated through shadow mask; (vi) Au electrode evaporated through shadow mask.


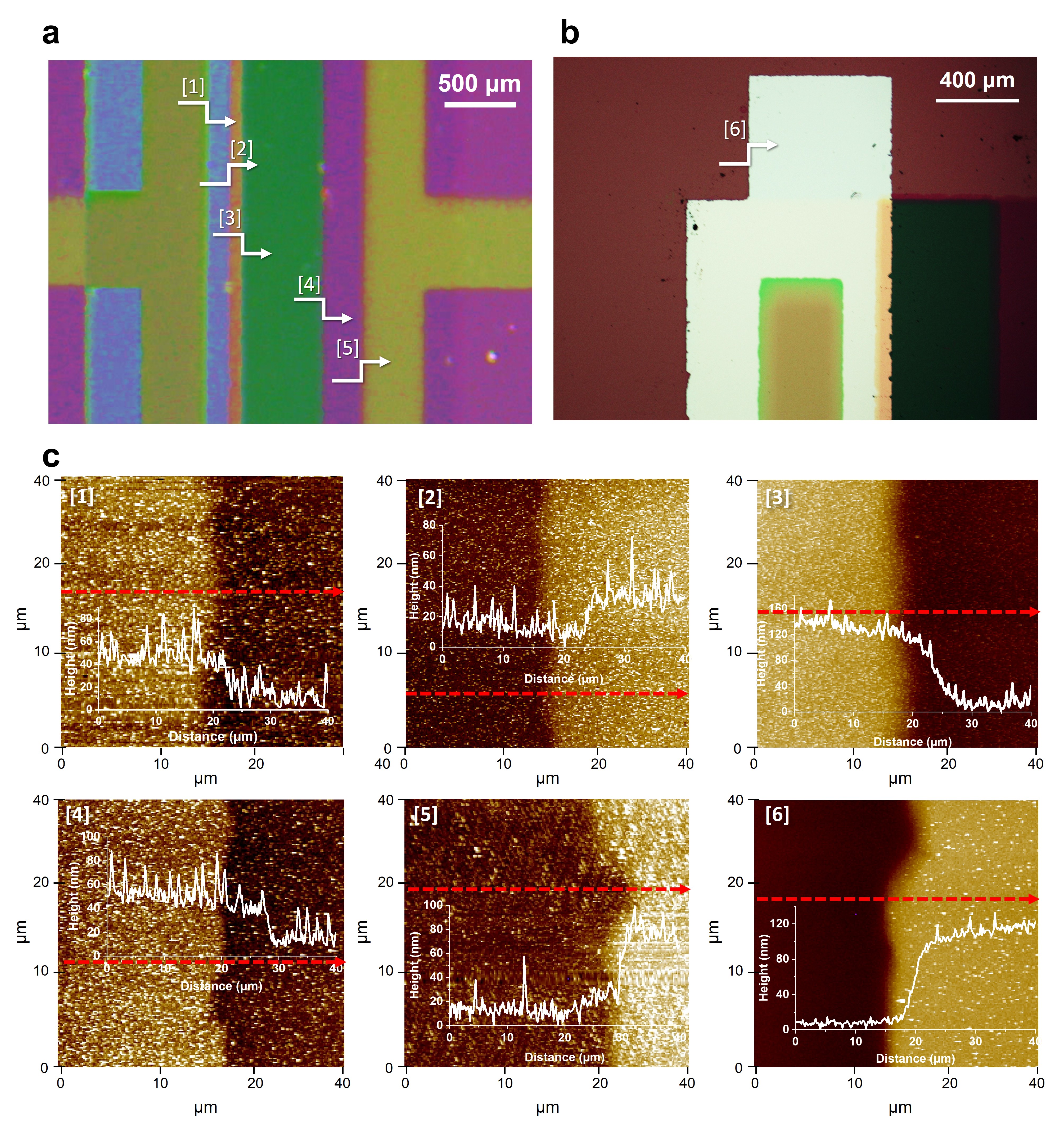


**Figure S2.** OM image of the AFM-measured region (a) and the screen-gate structure (b), and AFM step-height profiles (c) at [1] electrode/PH-BTBT-10, [2] PH-BTBT-10/heterojunction, [3] screen gate/heterojunction, [4] heterojunction/PTCDI-C13, and [5] PTCDI-C13/electrode, [6] parylene/screen gate.

**
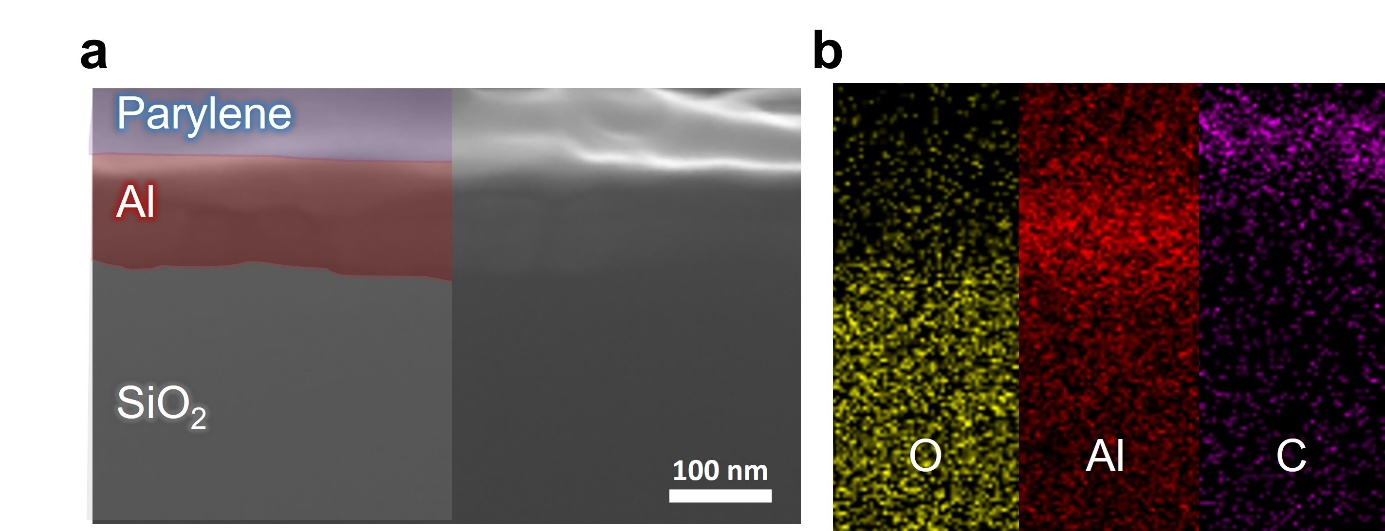
**

**Figure S3.** (a) Cross-sectional SEM image of the SiO_2_/screen gate/parylene layers measured to confirm the proper deposition of the screen gate between the dielectric layers. (b) EDS mapping image of screen gate structure cross-section view.

**
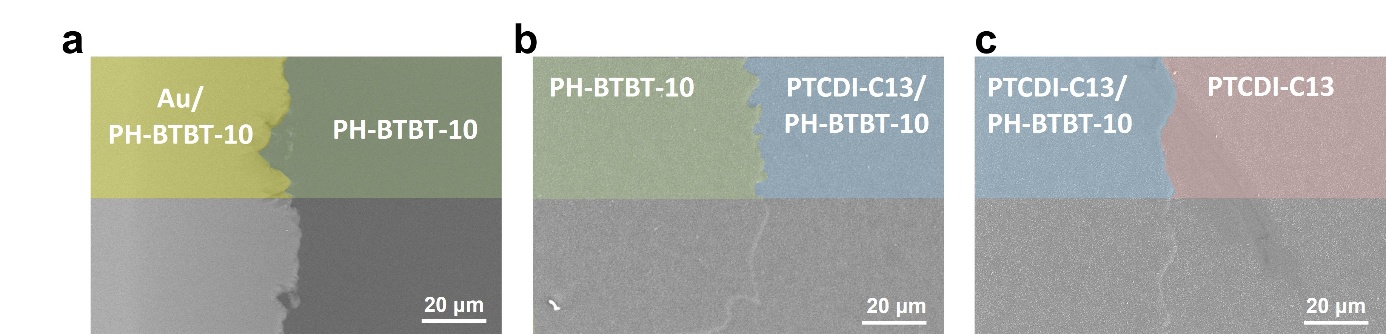
**

**Figure S4.** Surface SEM images of the GA-Transistor heterojunction. SEM images highlighting key material boundaries in the device: (a) The Au/PH-BTBT-10 to PH-BTBT-10 interface; (b) The PH-BTBT-10 to PTCDI-C13/PH-BTBT-10 heterojunction interface; (c) The PTCDI-C13/PH-BTBT-10 heterojunction to PTCDI-C13 interface

**
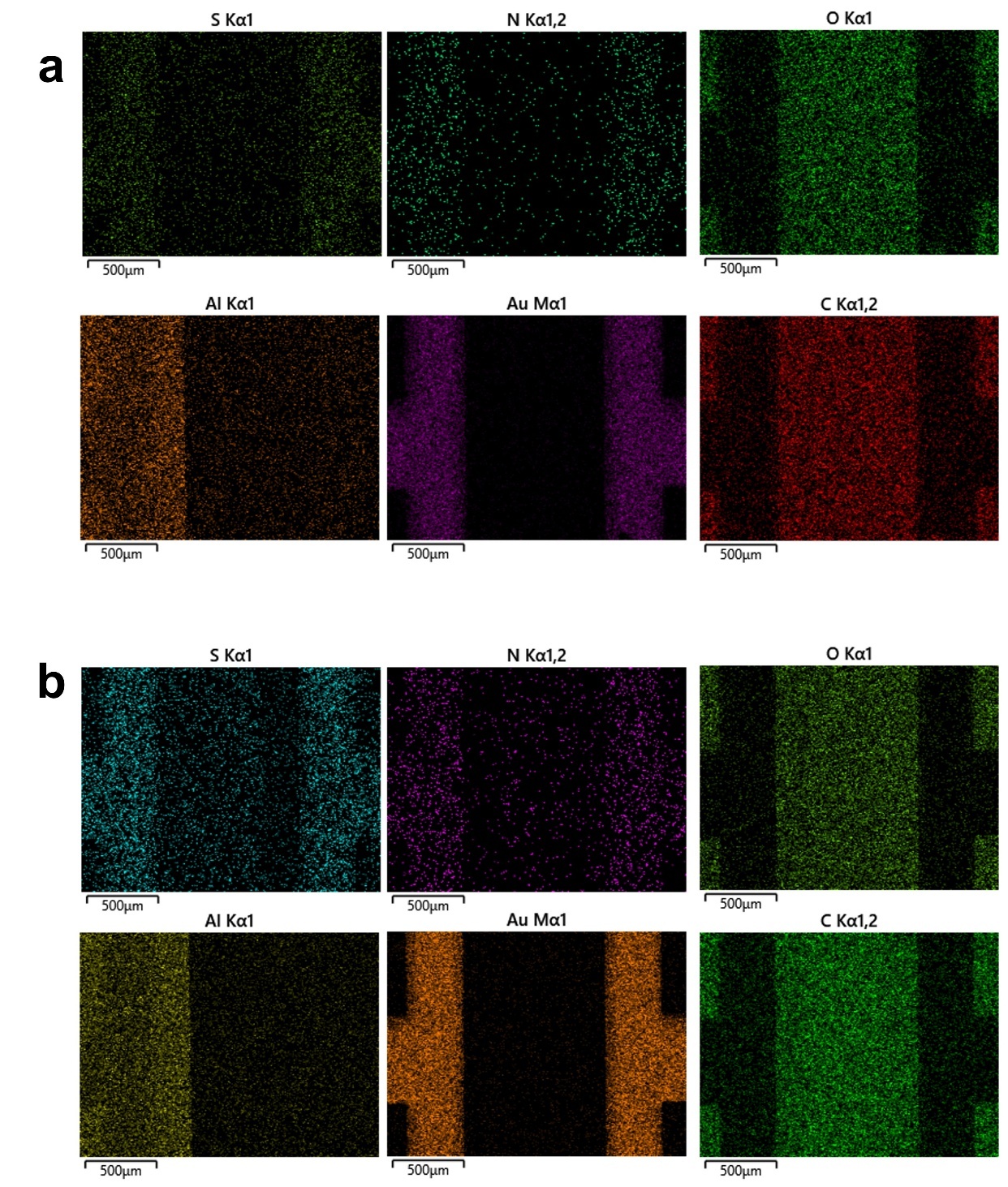
**

**Figure S5.** EDS mapping images providing compositional analysis of the device structures: (a) EDS mapping of the SA-transistor and (b) EDS mapping of the GA-transistor.


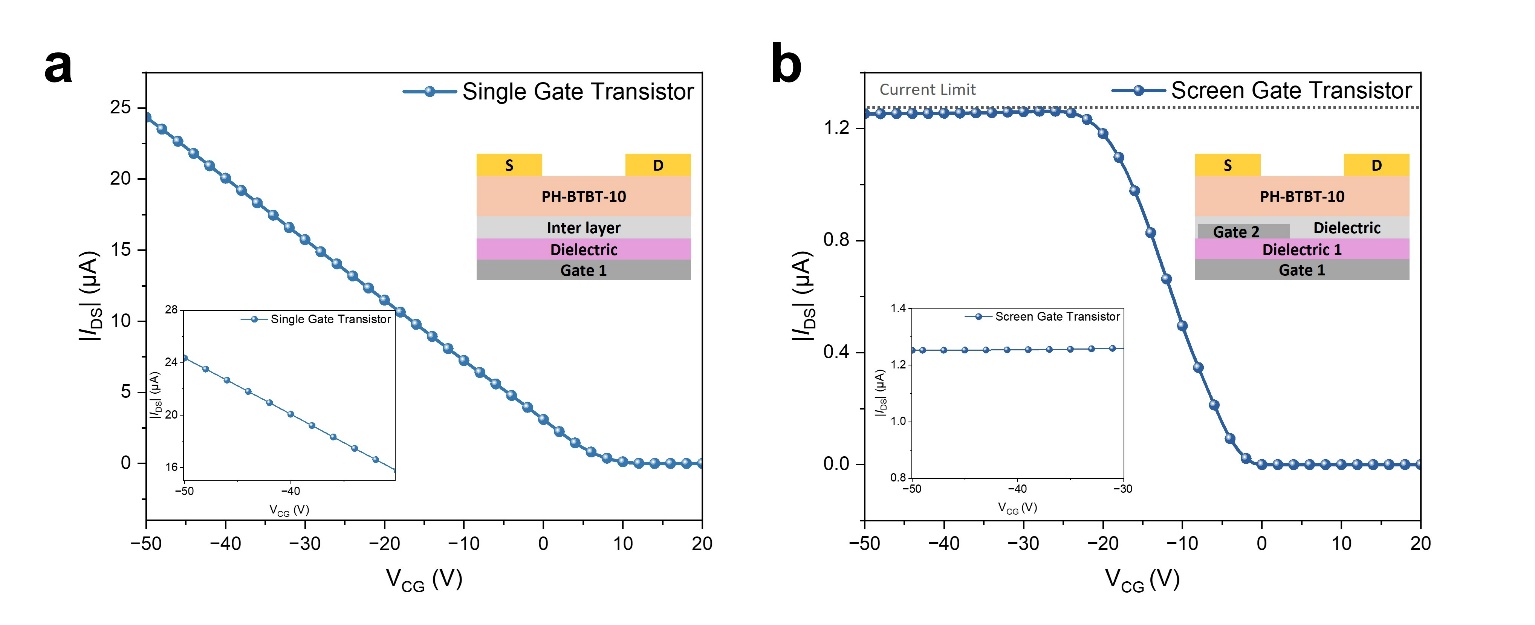


**Figure S6.** Transfer characteristics of (a) a conventional three-terminal transistor and (b) a screen gate-inserted SA-transistor.


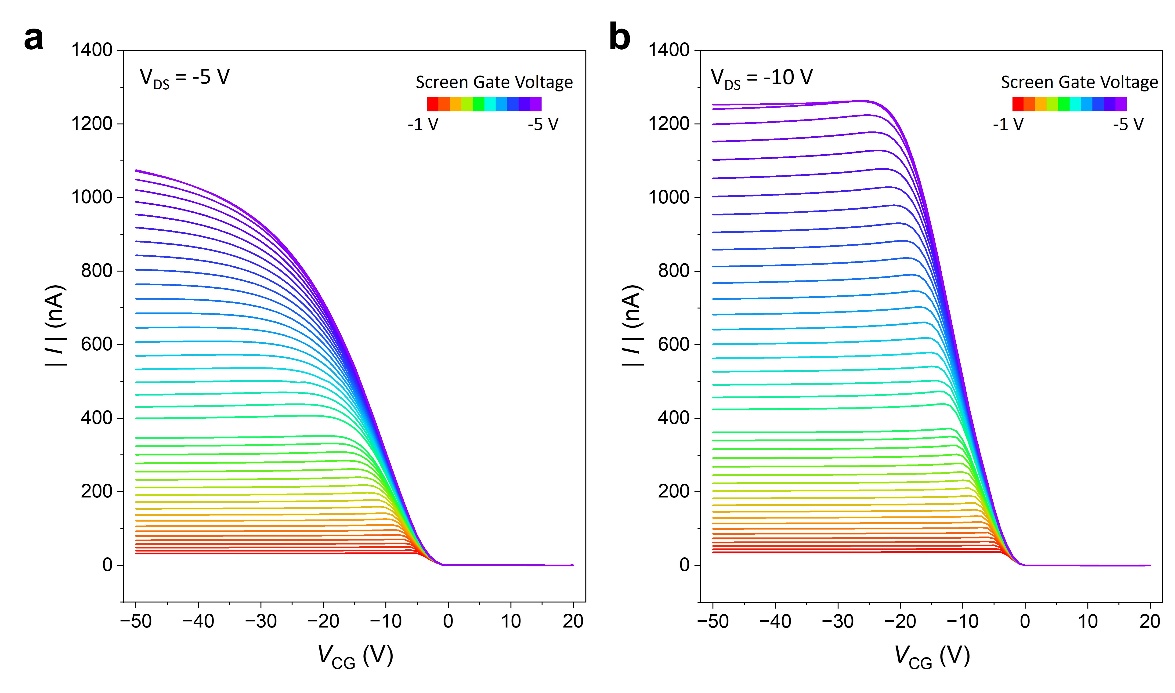


**Figure S7.** Transfer characteristics of the device with fixed *V*_DS_ of (a) −5 V and (b) −10 V. The *V*_Screen-G_ was varied from −1 V to −5 V with a step voltage of −0.1 V. The *V*_CG_ was swept from 20 V to −50 V.


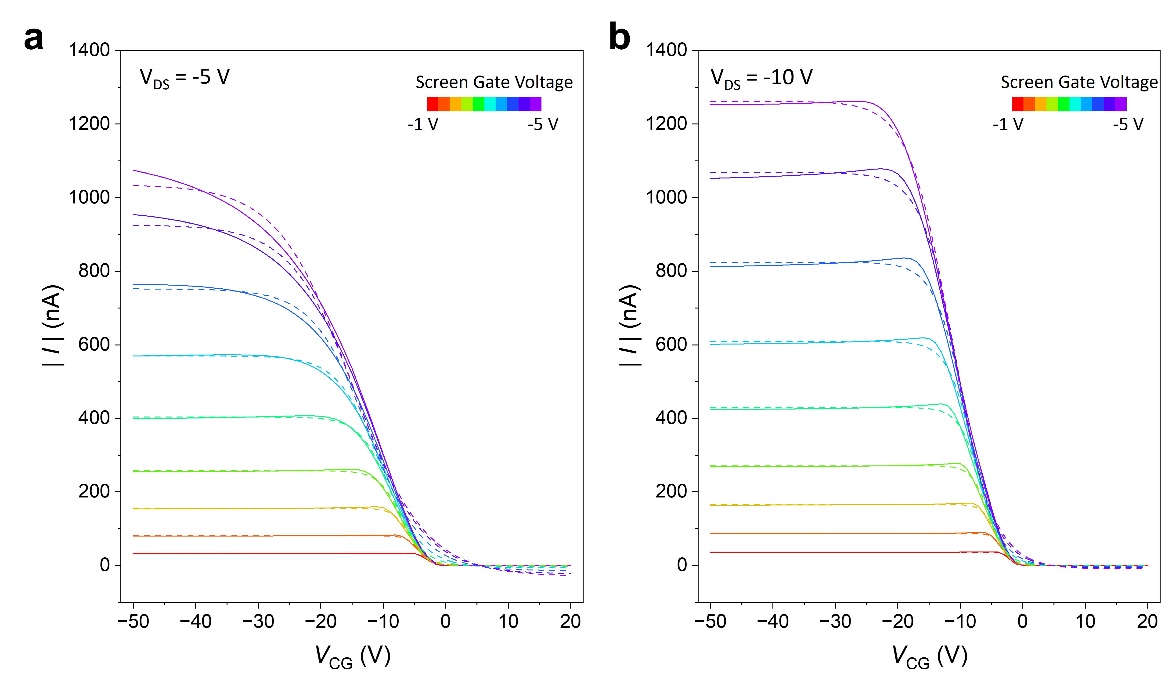


**Figure S8.** Sigmoid fitting results of the transfer characteristics extracted at intervals of −0.5 V within the *V*_Screen-G_ range from −1 V to −5 V: (a) at *V*_DS_ = −5 V, and (b) at *V*_DS_ = −10 V. Solid lines denote the measured data, and dashed lines represent the fitted sigmoid functions.


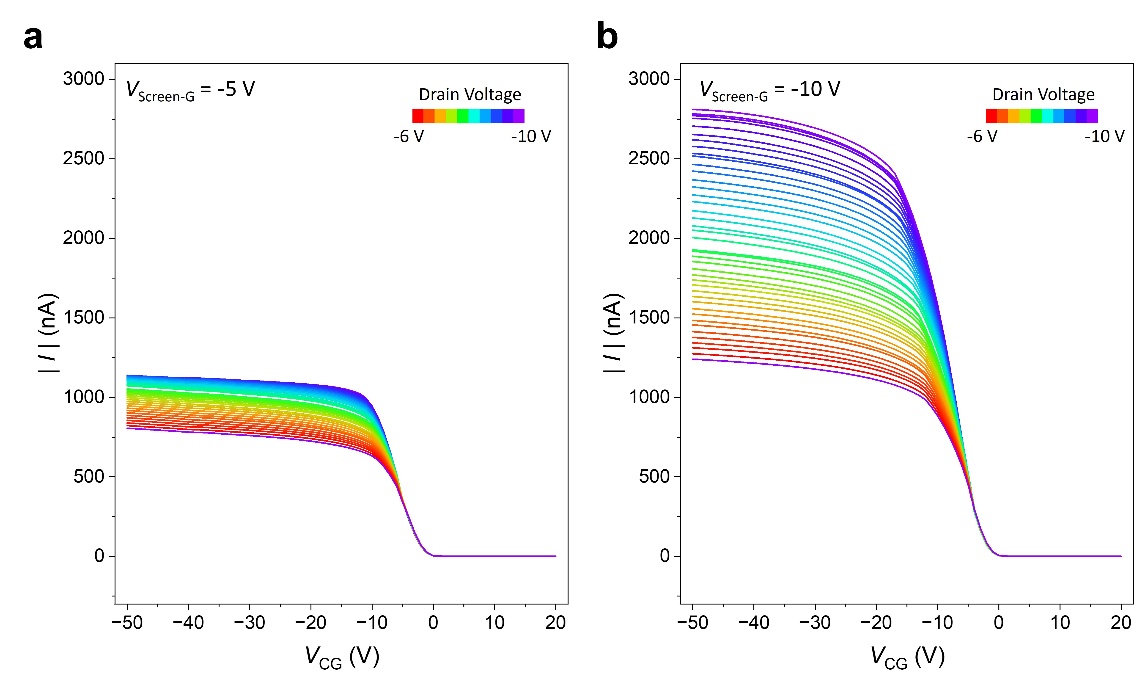


**Figure S9.** Transfer characteristics measured with *V*_DS_ varied from −6 V to −10 V in steps of −0.1 V. The *V*_Screen-G_ was fixed at (a) −5 V and (b) −10 V for each measurement. The *V*_CG_ was swept from 20 V to −50 V.

**
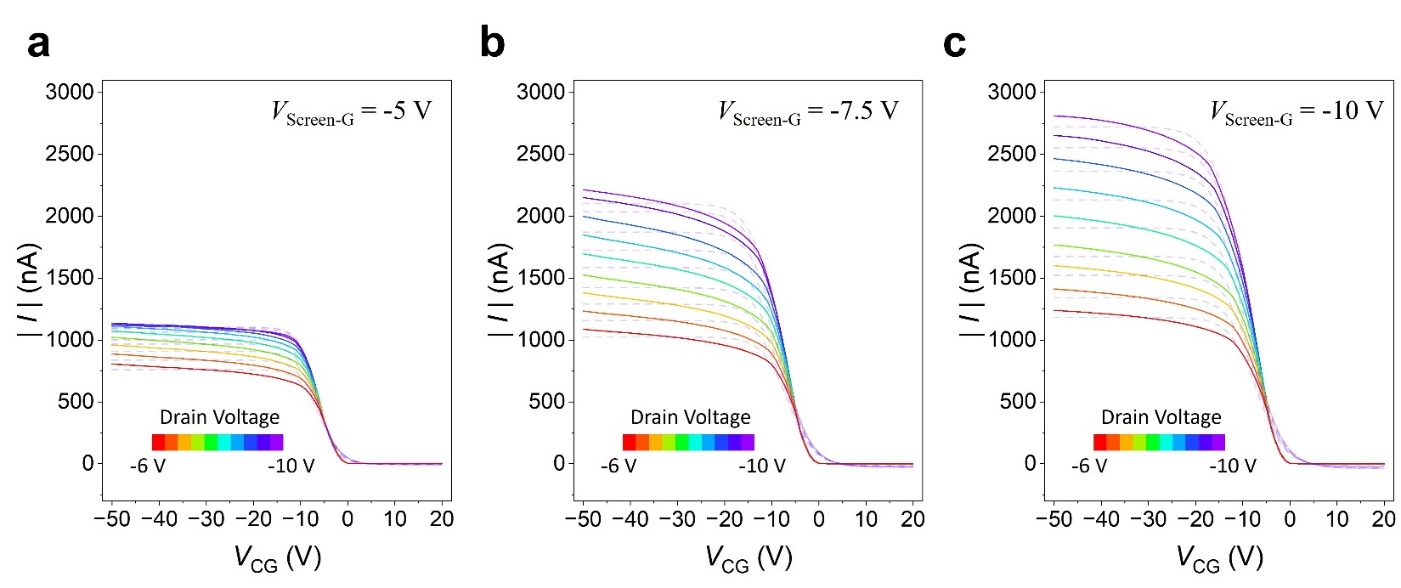
**

**Figure S10.** Sigmoid fitting results of the transfer characteristics extracted at intervals of −0.5 V within the *V*_DS_ range from −6 V to −10 V: (a) at *V*_Screen-G_ = −5 V, (b) at *V*_Screen-G_ = −7.5 V, and (c) at *V*_Screen-G_ = −10 V. Solid lines represent the measured data, while dashed lines indicate the fitted sigmoid curves.

**
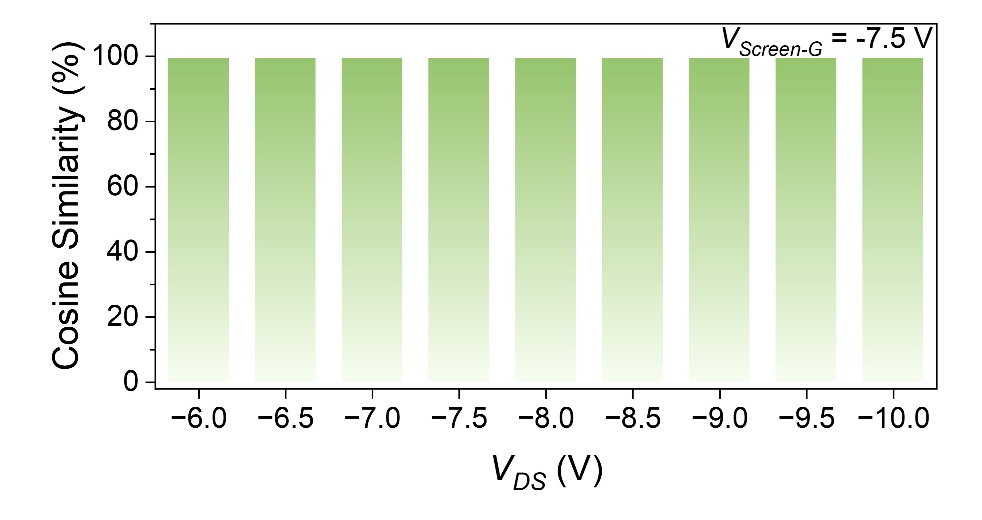
**

**Figure S11.** Cosine similarity between the measured transfer curves and the fitted sigmoid functions as a function of the *V*_DS_ varied from −6 V to −10 V.


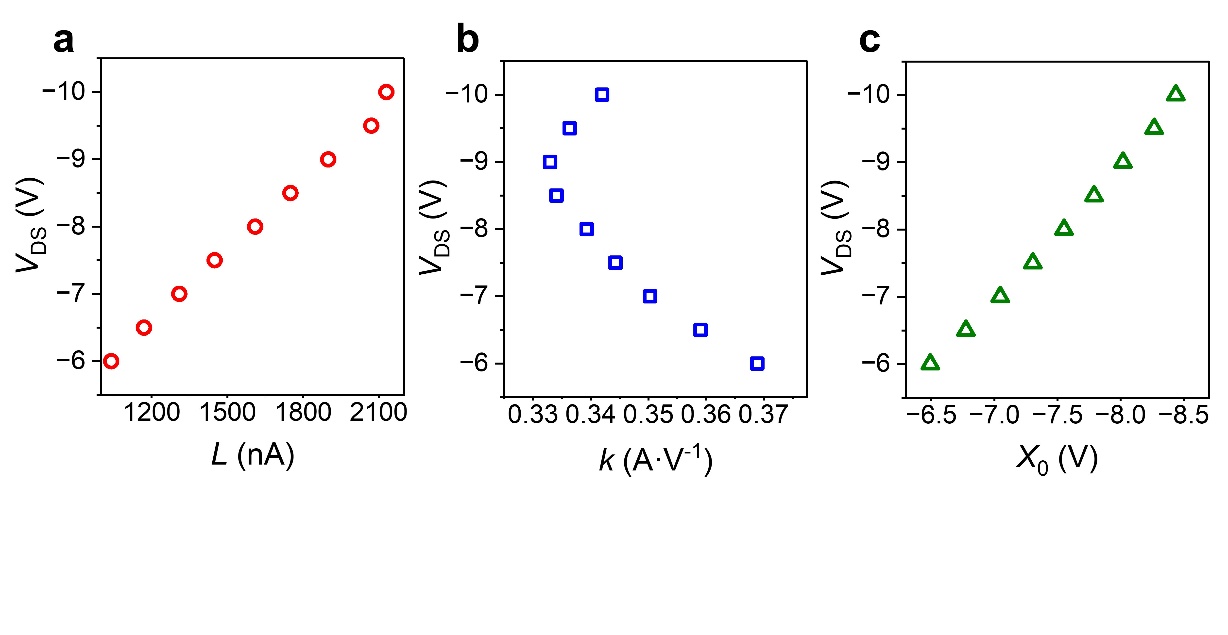


**Figure S12.** Extracted values of the sigmoid parameters (a) *L*, (b) *k*, and (c) *X*_0_ as a function of the *V*_DS_ varied from −6 V to −10 V.


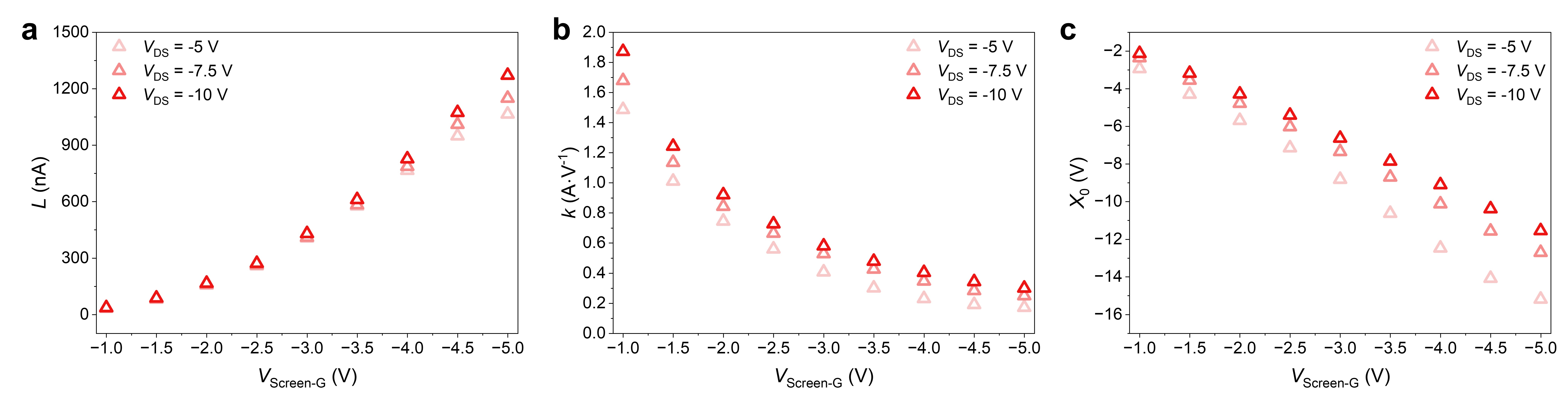


**Figure S13.** Extracted values of the sigmoid parameters (a) *L*, (b) *k*, and (c) *X*_0_ as a function of the *V*_Screen-G_ varied from −1 V to −5 V, when *V*_DS_ was −5, −7.5, and −10 V, respectively.

**
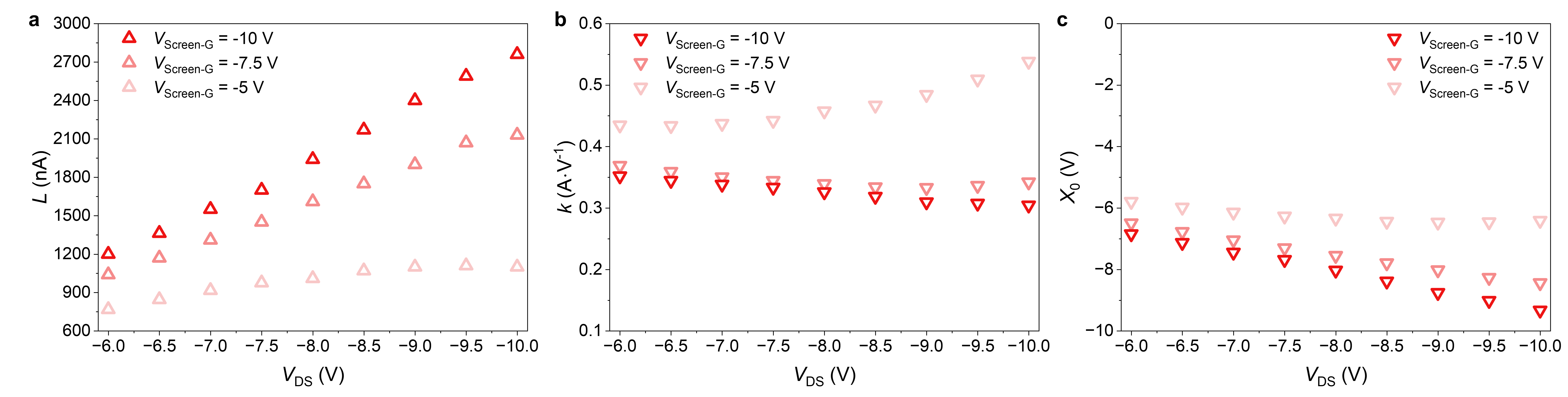
**

**Figure S14.** Extracted values of the sigmoid parameters (a) *L*, (b) *k*, and (c) *X*_0_ as a function of the *V*_DS_ varied from −6 V to −10 V, when *V*_Screen-G_ was −5, −7.5, and −10 V, respectively.


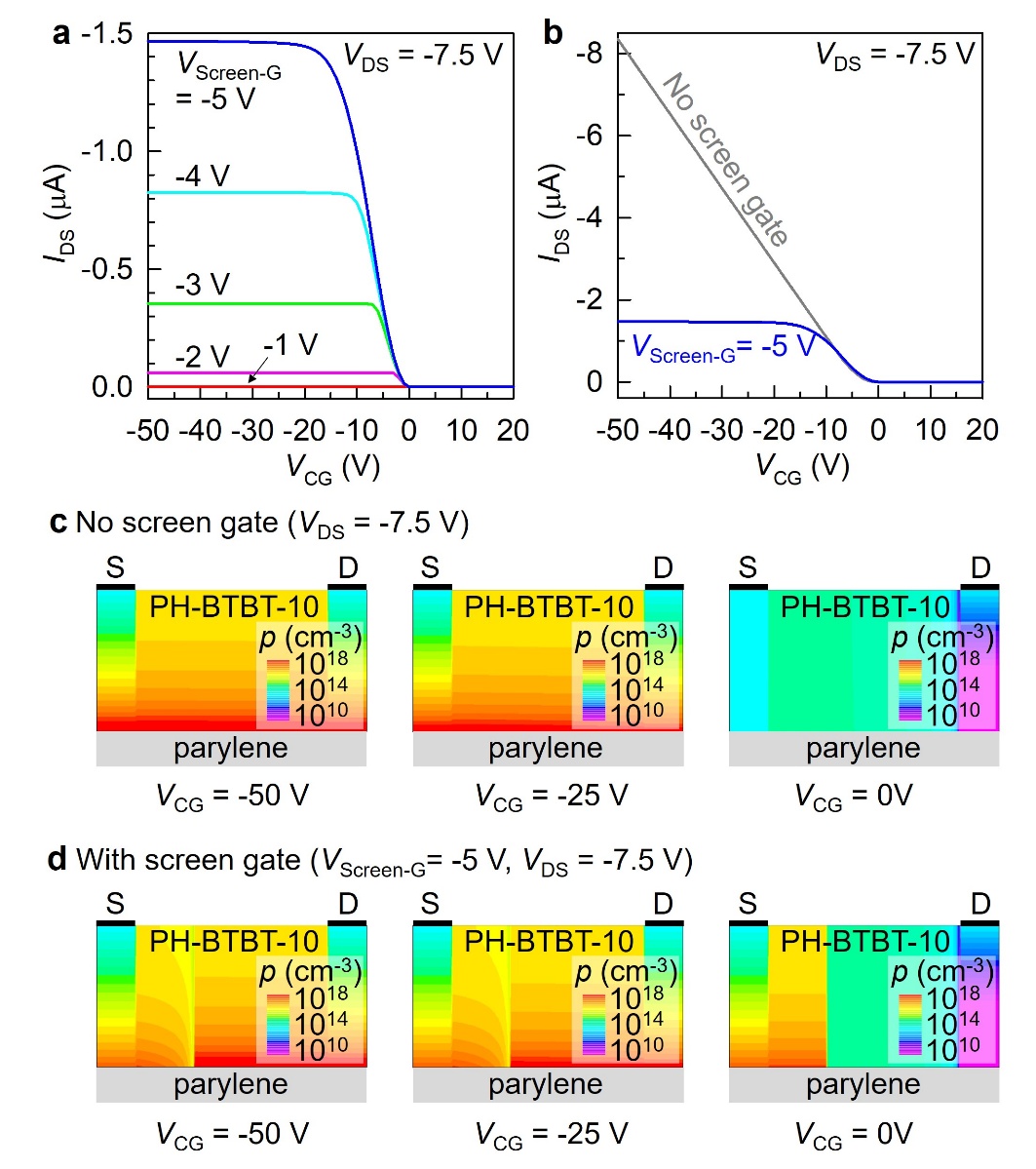


**Figure S15.** Drift-diffusion simulation on the fundamental mechanism of the SA-transistor. (a) Sigmoid-like transfer curve and its *V*_Screen-G_ control. (b) Comparison between the devices with and without the screen gate. (c) 2D hole distribution in the no-screen gate device. (d) 2D hole distribution in the sigmoid-like device (S: source, D: Drain).


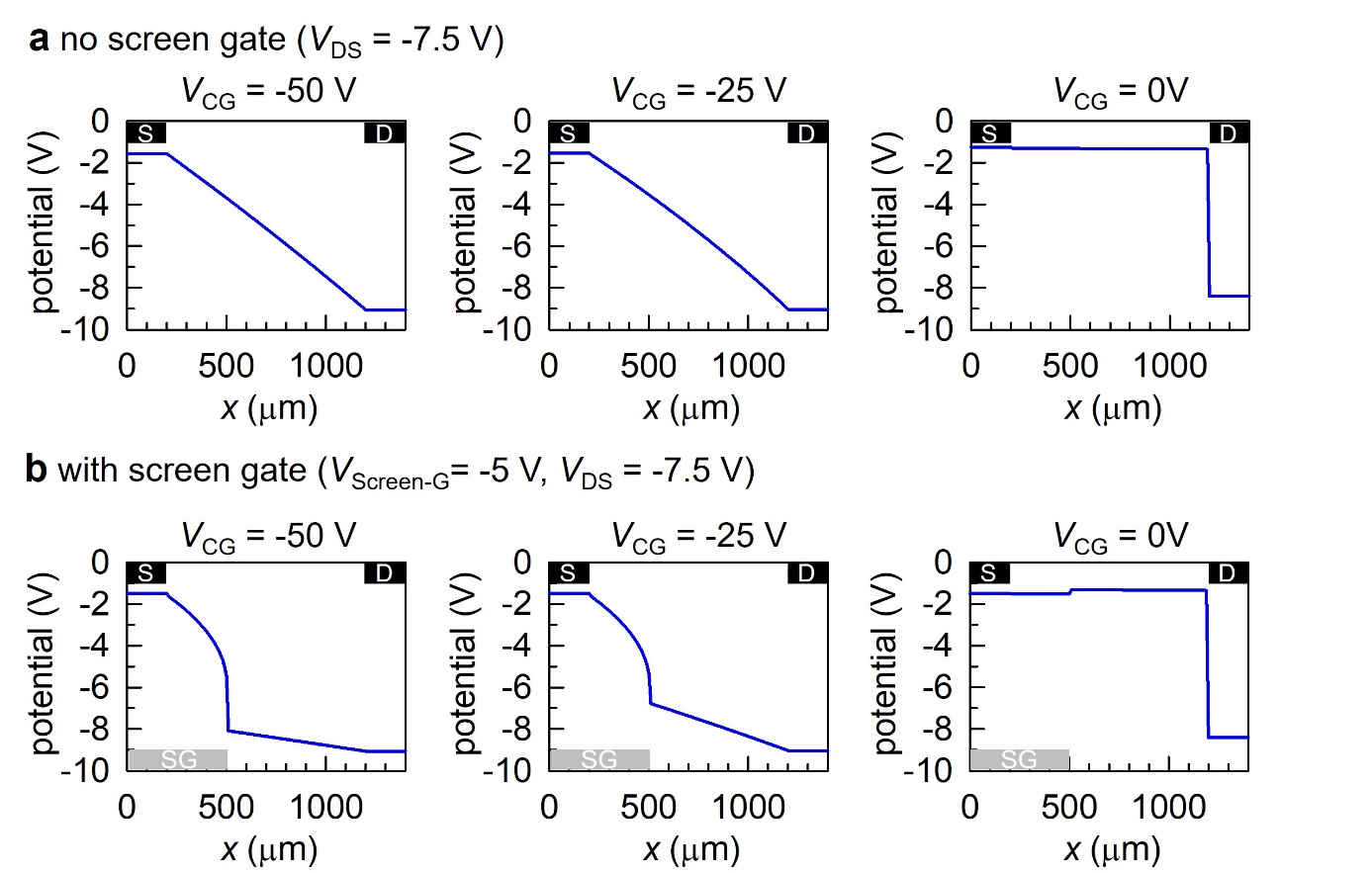


**Figure S16.** 1D potential profiles extracted (a) w/o a screen gate and (b) w/ a screen gate (SA-transistor), matching the 2D hole distribution maps according to the common gate bias (*V*_CG_). These profiles were extracted 1 nm above the PH-BTBT-10/parylene interface to capture the region of carrier accumulation and surface charge transport. The vertical alignment with the source (S), drain (D), and screen gate (SG) electrodes in each device is shown as a visual guide.

**
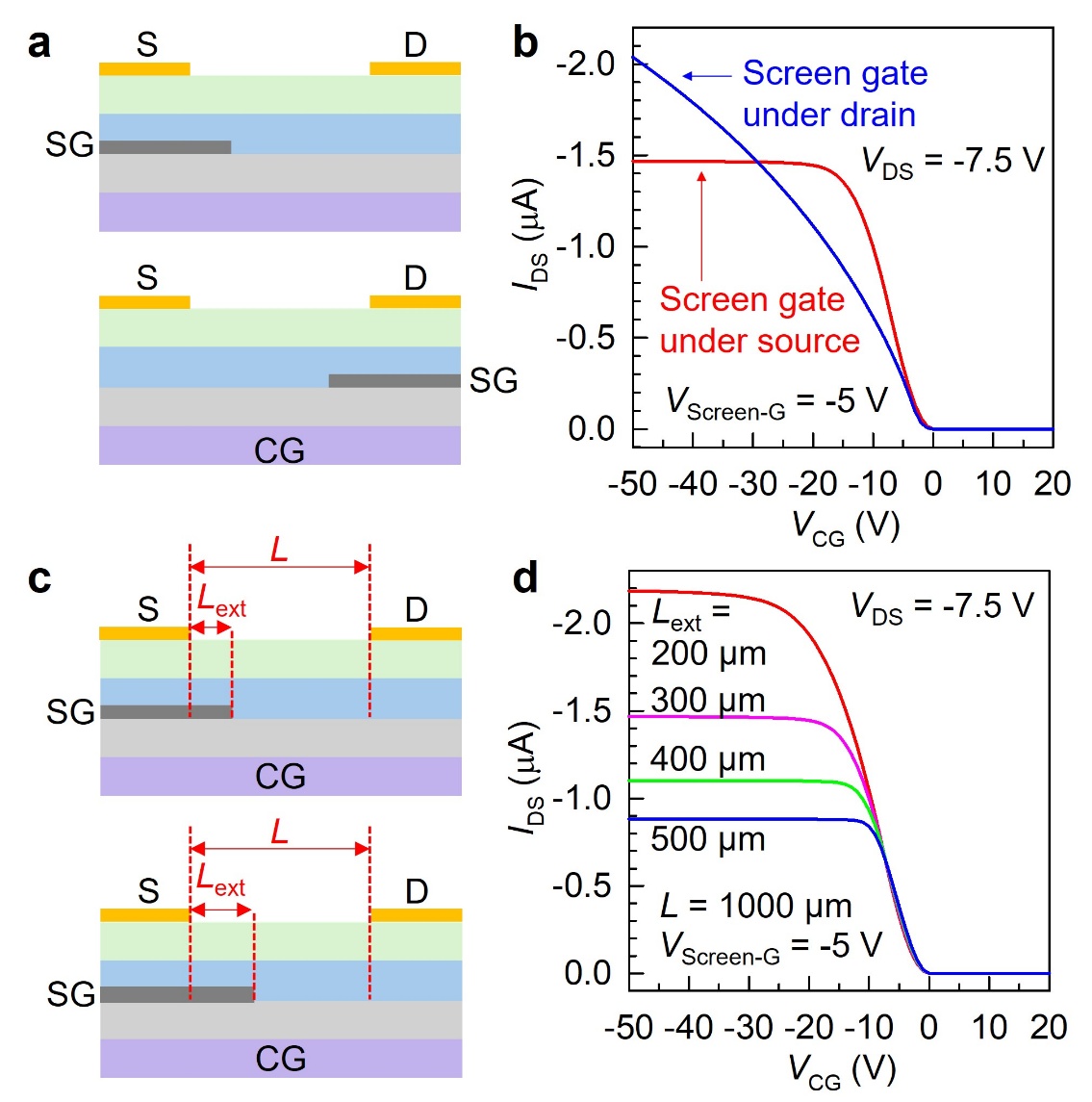
**

**Figure S17.** Drift-diffusion simulation on the structural aspect of the SA-transistor. (a, b) Illustration and results on the effects of the position of the screen gate. (c, d) Illustration and results on the effects of the screen gate *L*_ext_ (S: source, D: drain, CG: common gate, SG: screen gate).


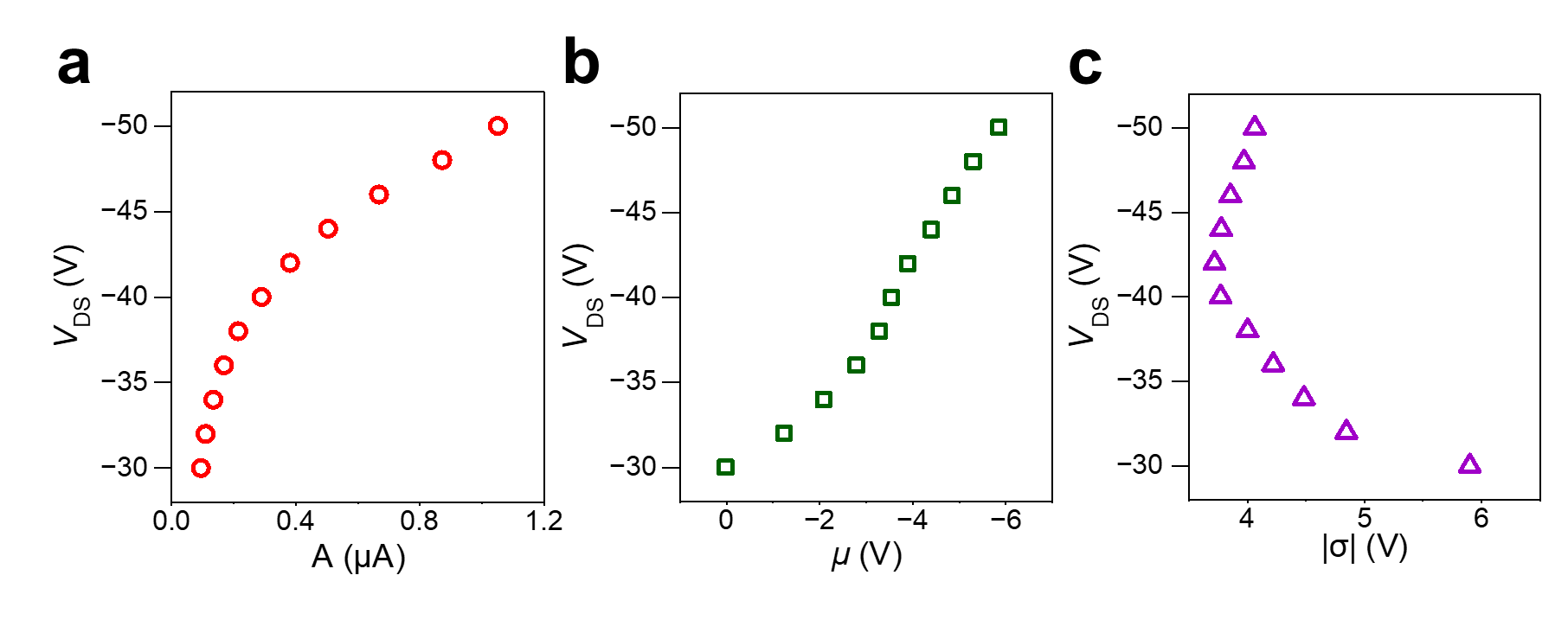


**Figure S18.** Extracted values of the Gaussian parameters (a) *A*, (b) *µ*, and (c) *σ* as a function of the *V*_DS_ varied from −30 V to −50 V.


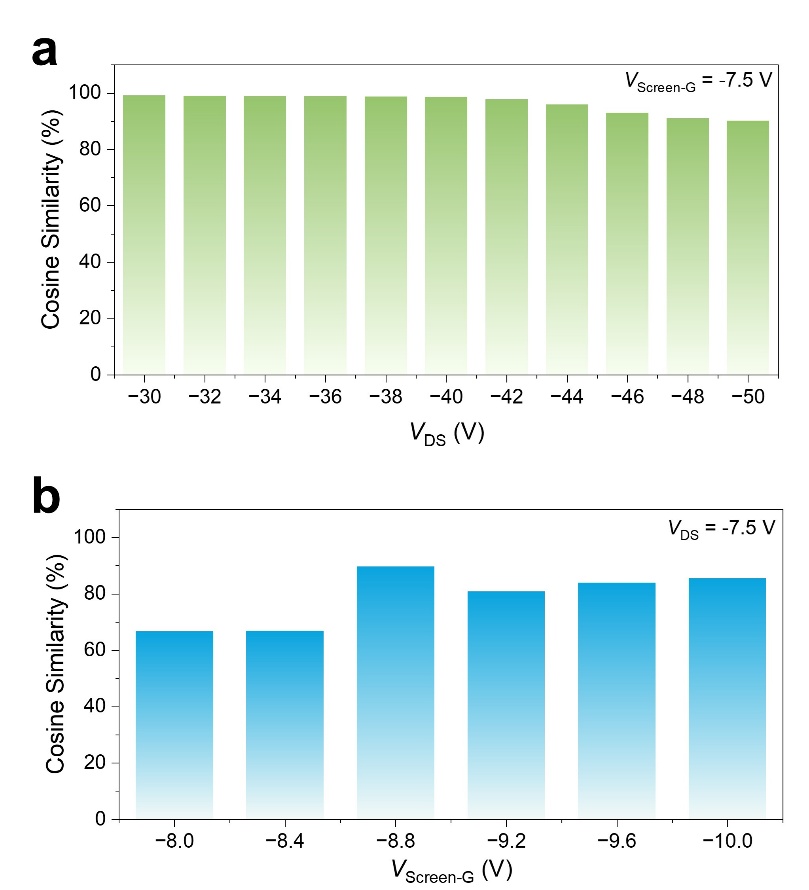


**Figure S19.** Cosine similarity between the measured transfer curves and the fitted Gaussian functions as a function of (a) the *V*_DS_ varied from −30 V to −50 V, and (b) the *V*_Screen-G_ varied from −8 V to −10 V.


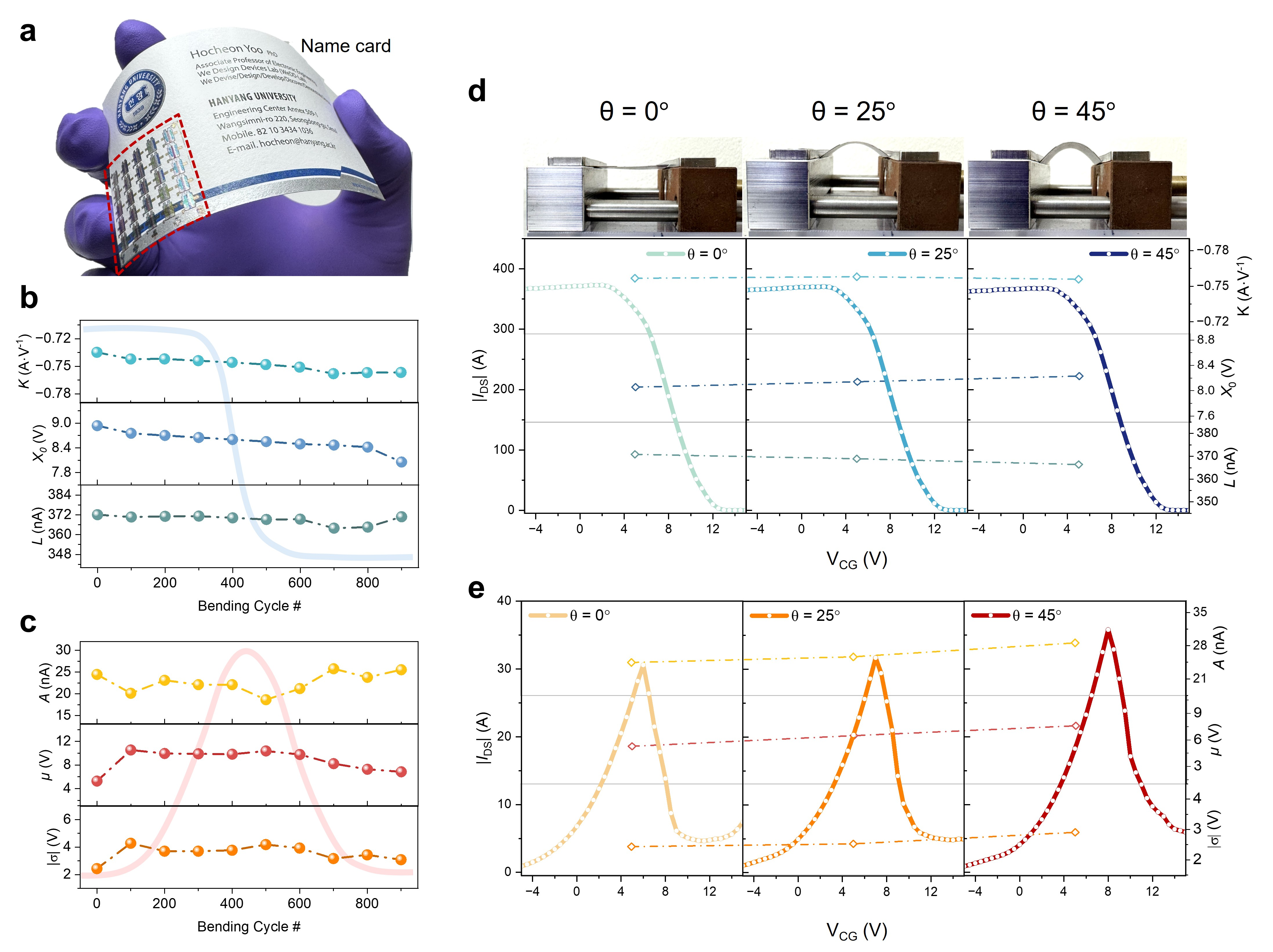


**Figure S20.** (a) The real image of SA-transistors and GA-transistors fabricated on a name card substrate. (b) Stability of key sigmoid parameters (*K*, *X*_0_, *L*) of the SA-transistor and (c) GA-transistor under 900 bending cycles (θ = 25°). (d) Transfer characteristics of the SA-transistor and (e) GA-transistor under different angles (θ = 0°, 25°, 45°).

**
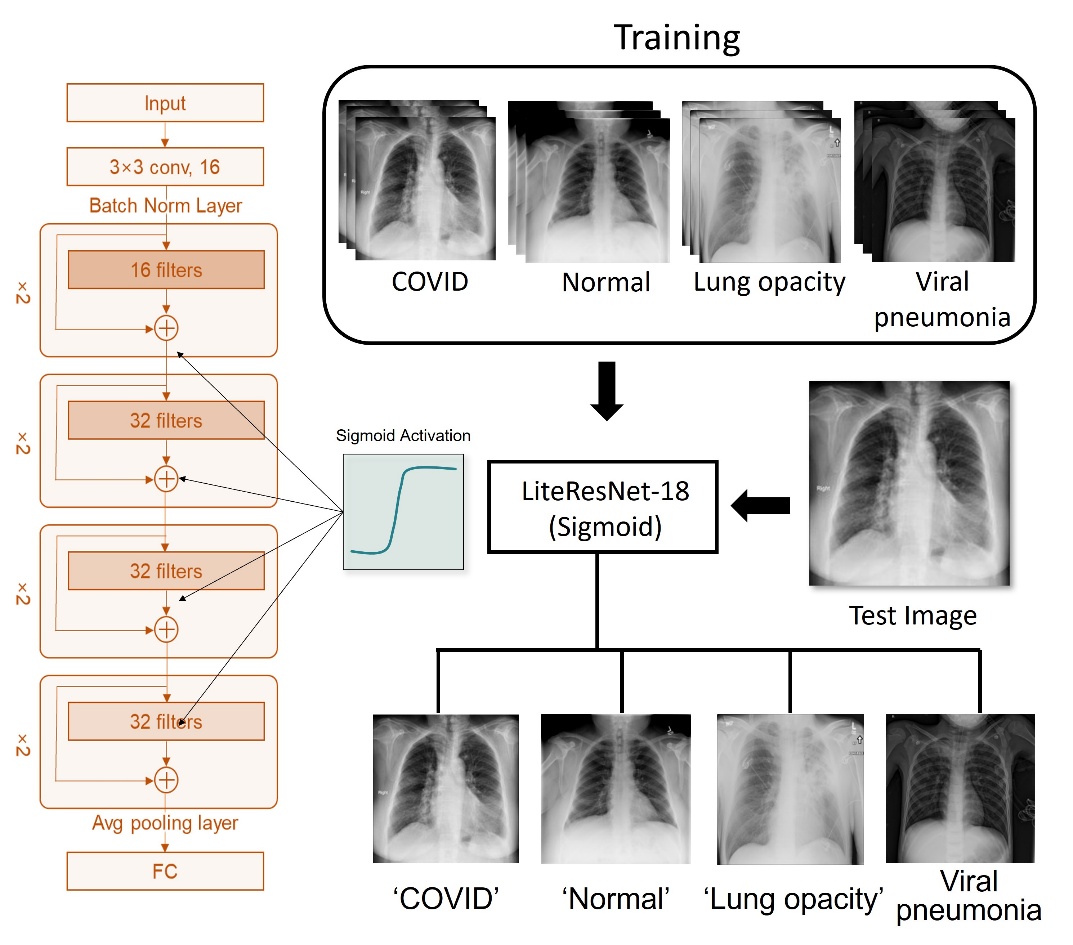
**

**Figure S21.** Architecture of the LiteResNet-18 neural network used for evaluating various activation functions. Comparison of classification accuracy across training epochs for networks using fixed Gaussian, fixed sigmoid, and tunable sigmoid activations. The tunable sigmoid activation demonstrates superior performance, achieving approximately 84% accuracy.


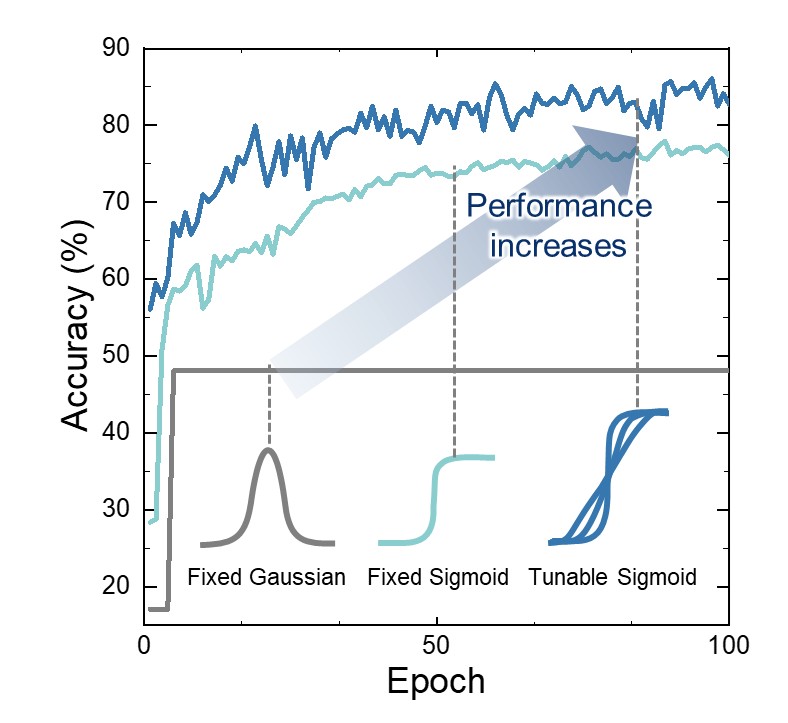


**Figure S22.** Comparison of classification accuracy across training epochs for networks using fixed Gaussian, fixed sigmoid, and tunable sigmoid activations. The tunable sigmoid activation demonstrates superior performance, achieving approximately 84% accuracy.

**
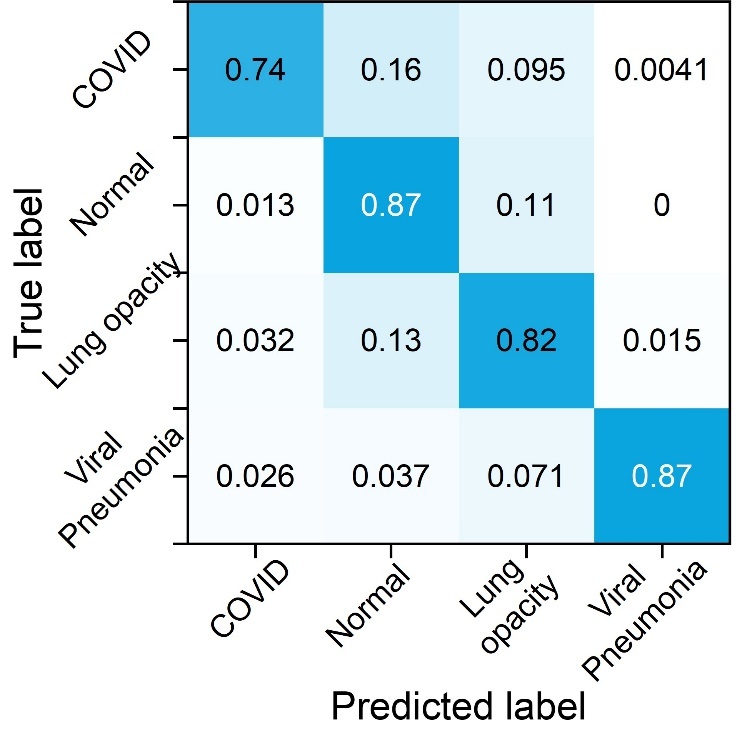
**

**Figure S23.** Confusion matrix analysis of MRI image classification. Confusion matrix illustrating classification performance of the LiteResNet-18 neural network with tunable sigmoid activation function for lung MRI image classification.

**
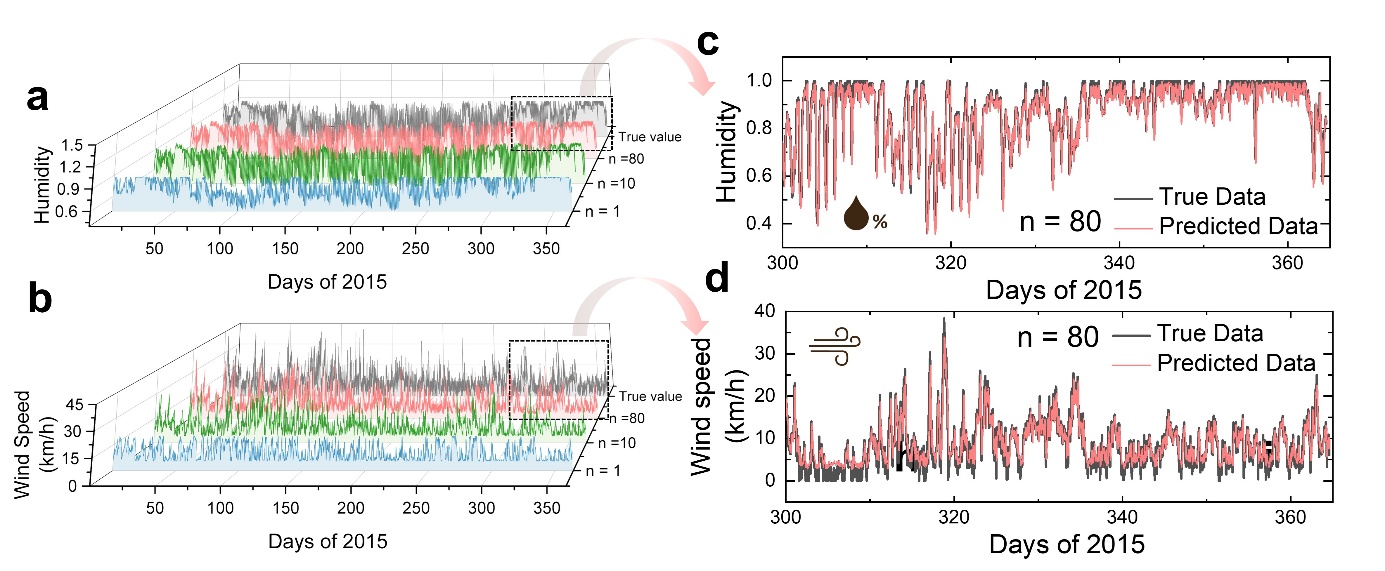
**

**Figure S24.** (a, b) Predicted (colored lines) versus actual (black solid line) environmental measurements for (a) humidity, and (b) wind speed, showing enhanced predictive accuracy as the number of Gaussian kernels increases (blue: 1 kernel, green: 10 kernels, red: 80 kernels). (c, d) Direct comparison between actual (x-axis) and predicted (y-axis) values for (c) humidity, and (d) wind speed at the highest kernel count (*N*=80), highlighting excellent prediction accuracy.

**
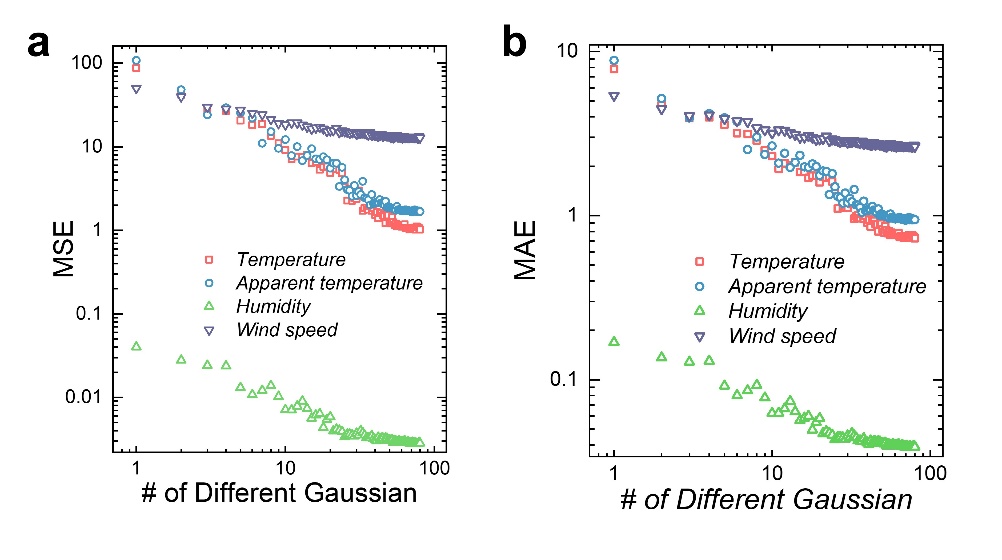
**

**Figure S25.** Evaluation of prediction accuracy as a function of Gaussian kernels. (a) MSE versus the number of Gaussian kernels (b) MAE versus the number of Gaussian kernels.


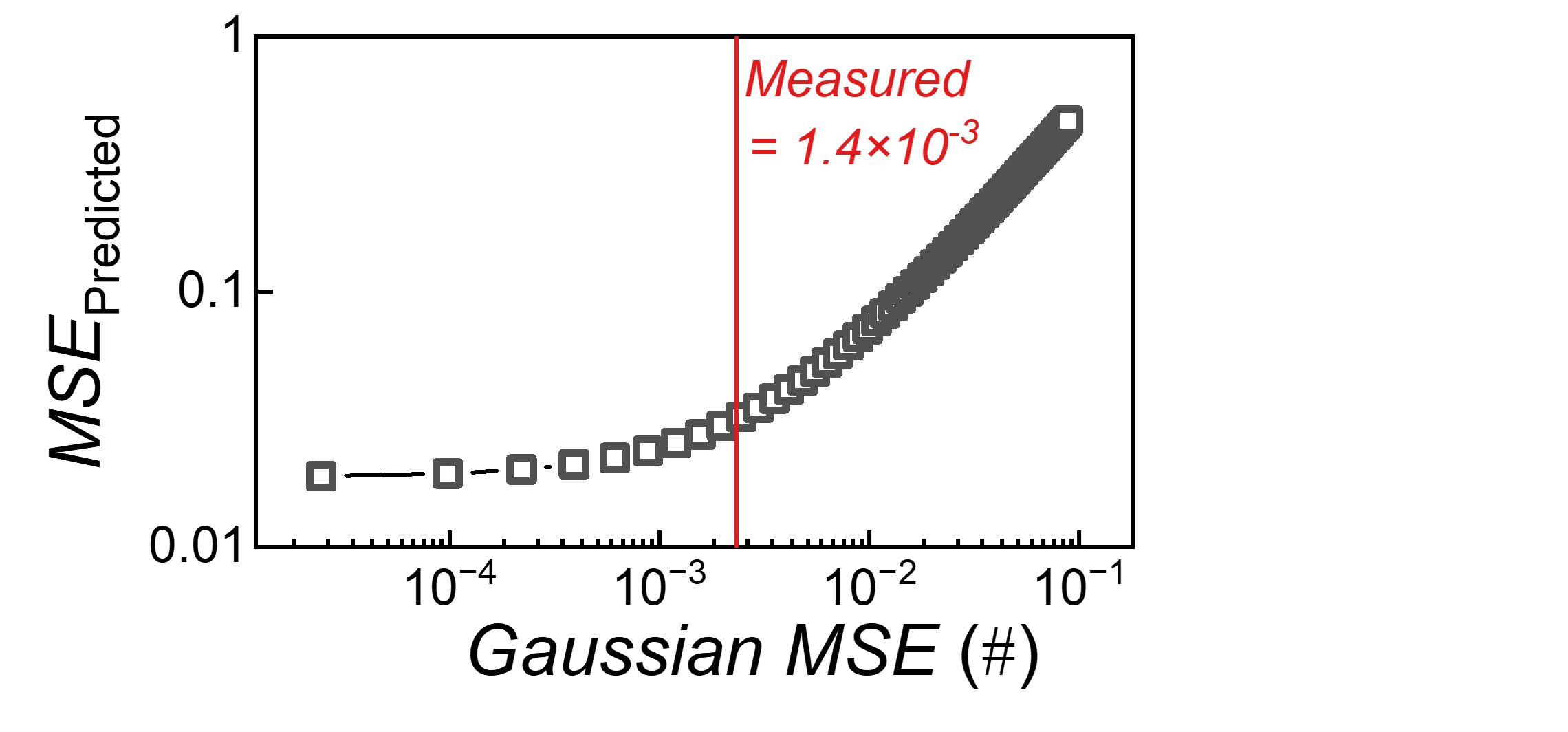


**Figure S26.** Analysis of how the distortion (MSE) in Gaussian kernels, reflecting non-ideal device characteristics, affects the prediction accuracy (MSE of predicted signals) in the RBF network simulations.


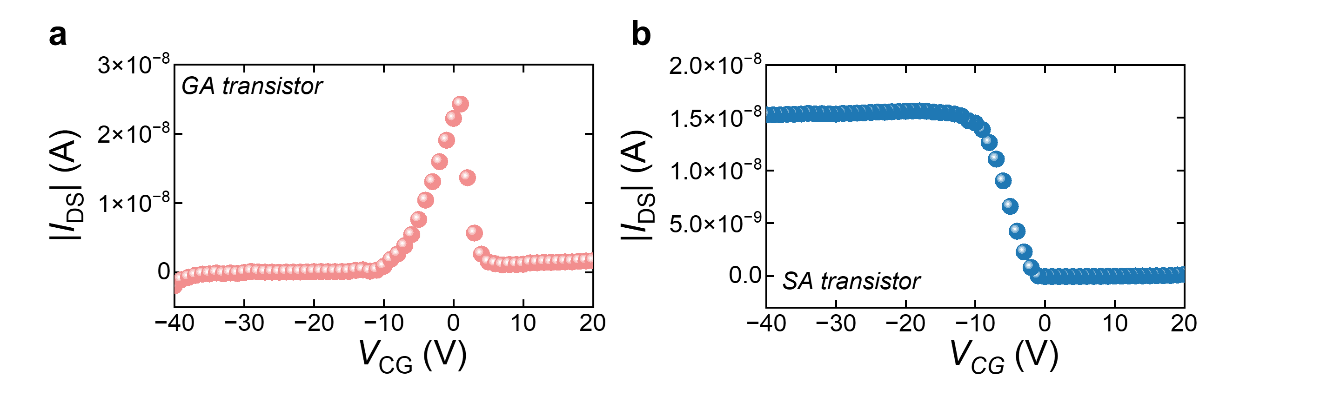


**Figure S27.** (a) Transfer curve of the GA-transistor. (b) Transfer curve of the SA-transistor.


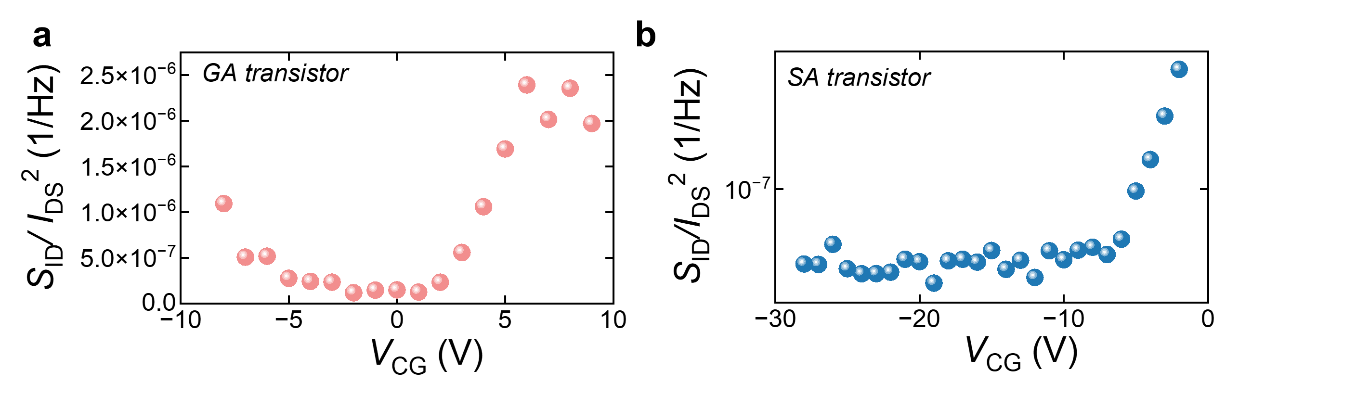


**Figure S28.** (a) *S*_ID_/*I*_D_² versus *V*_CG_ of the GA-transistor. (b) *S*_ID_/*I*_D_² versus *V*_CG_ of the SA-transistor.


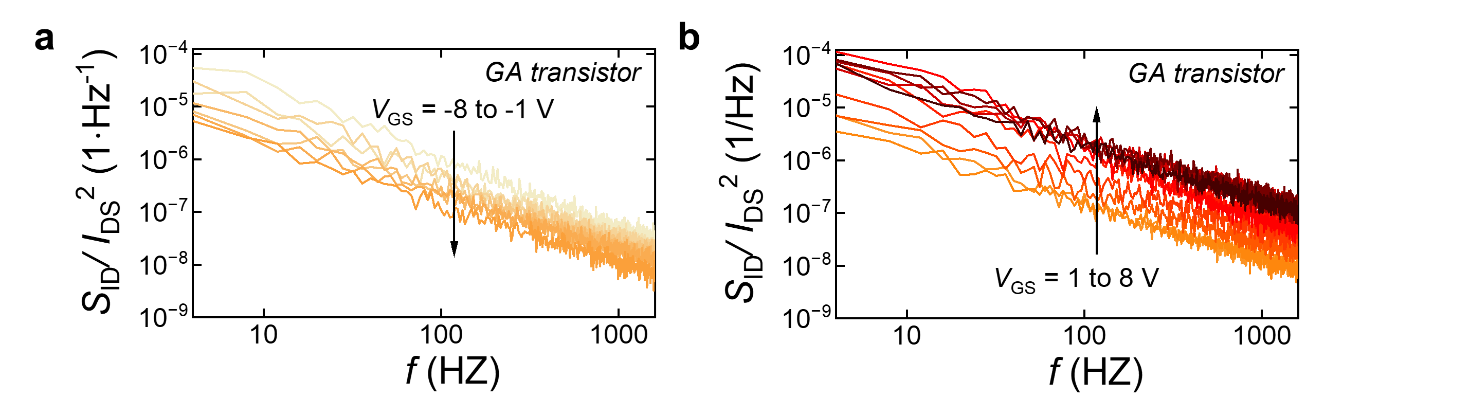
**Figure S29.** (a) *S*_ID_/*I*_D_^2^ versus frequency of the GA-transistor at *V*_CG_ = −8 to −1 V. (b) *S*_ID_/*I*_D_^2^ versus frequency of the GA-transistor at *V*_CG_ = +1 to +8 V.


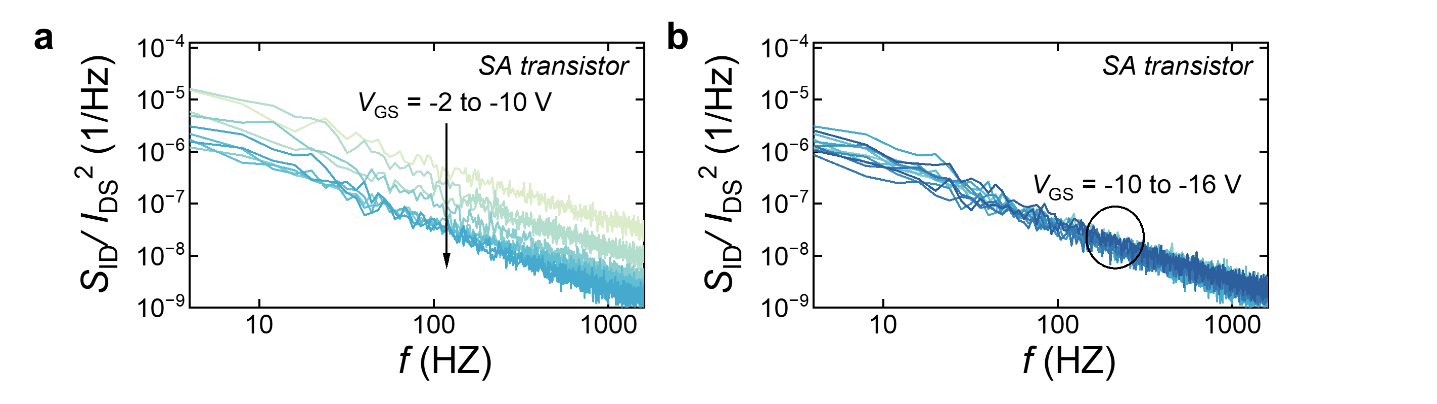


**Figure S30.** (a) *S*_ID_/*I*_D_² versus frequency of the SA-transistor at *V*_CG_ = −2 to −10 V. (b) *S*_ID_/*I*_D_² versus frequency of the SA-transistor at *V*_CG_ = −10 to −16 V.


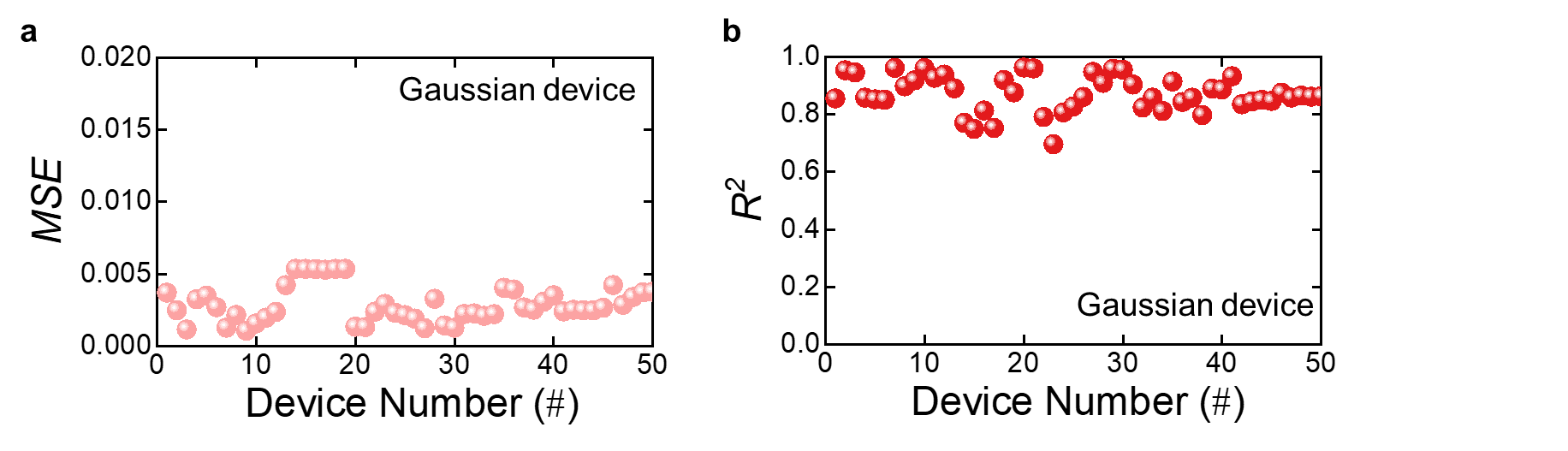


**Figure S31.** Measured non-ideality across 50 Gaussian activation transistor devices expressed as (a) MSE and (b) *R*^2^.


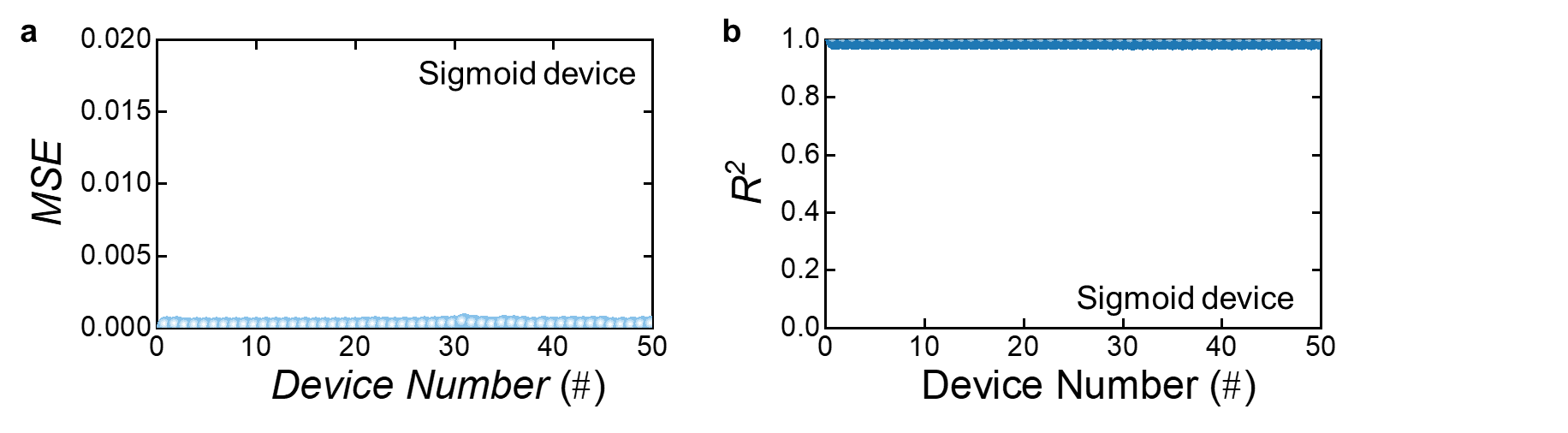


**Figure S32.** Measured non-ideality across 50 sigmoid activation transistor devices expressed as (a) MSE and (b) *R*^2^.


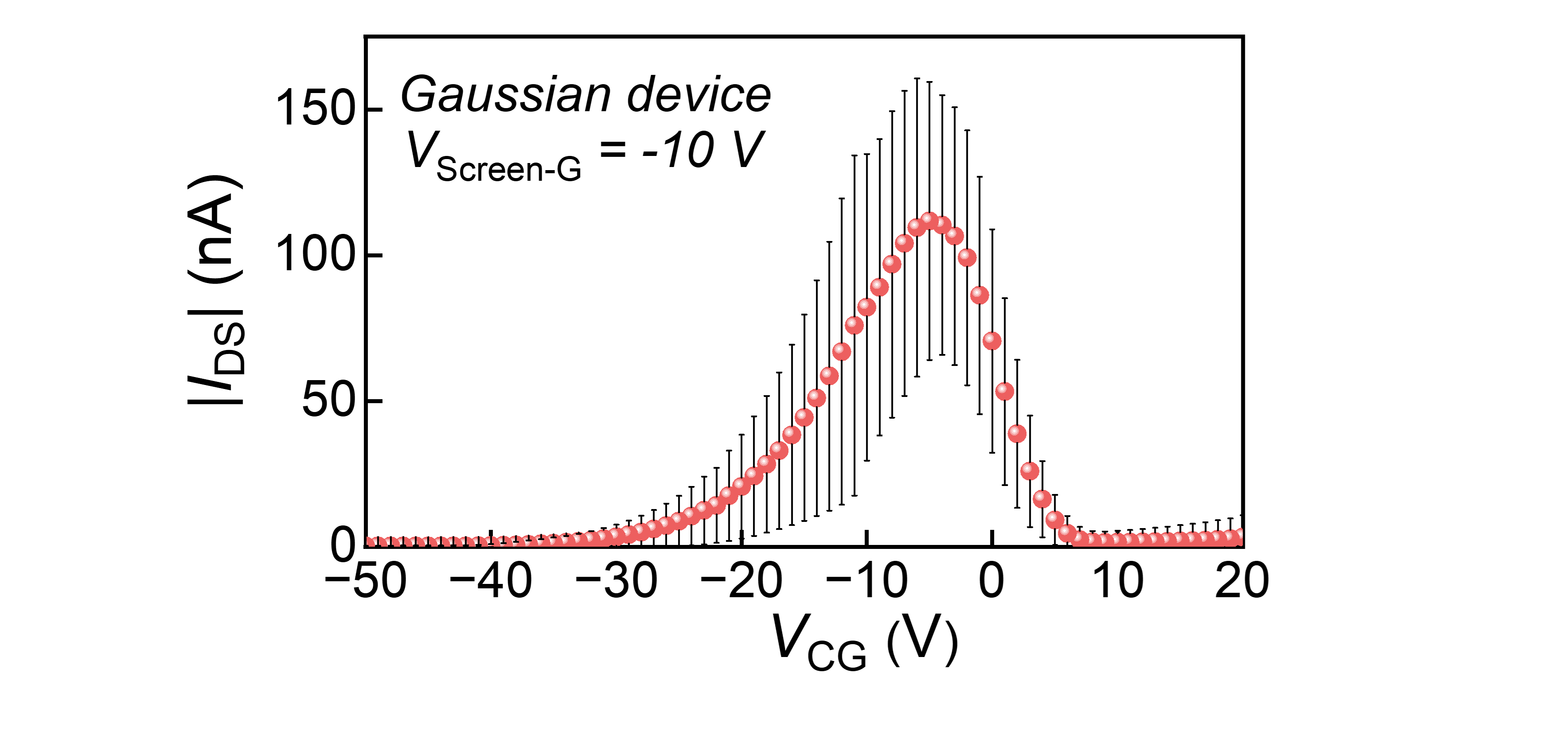


**Figure S33.** Transfer curves (*I*_DS_ versus *V*_CG_) for 50 measured sigmoid activation devices, showing the mean curve and error bars representing the standard deviation across devices.


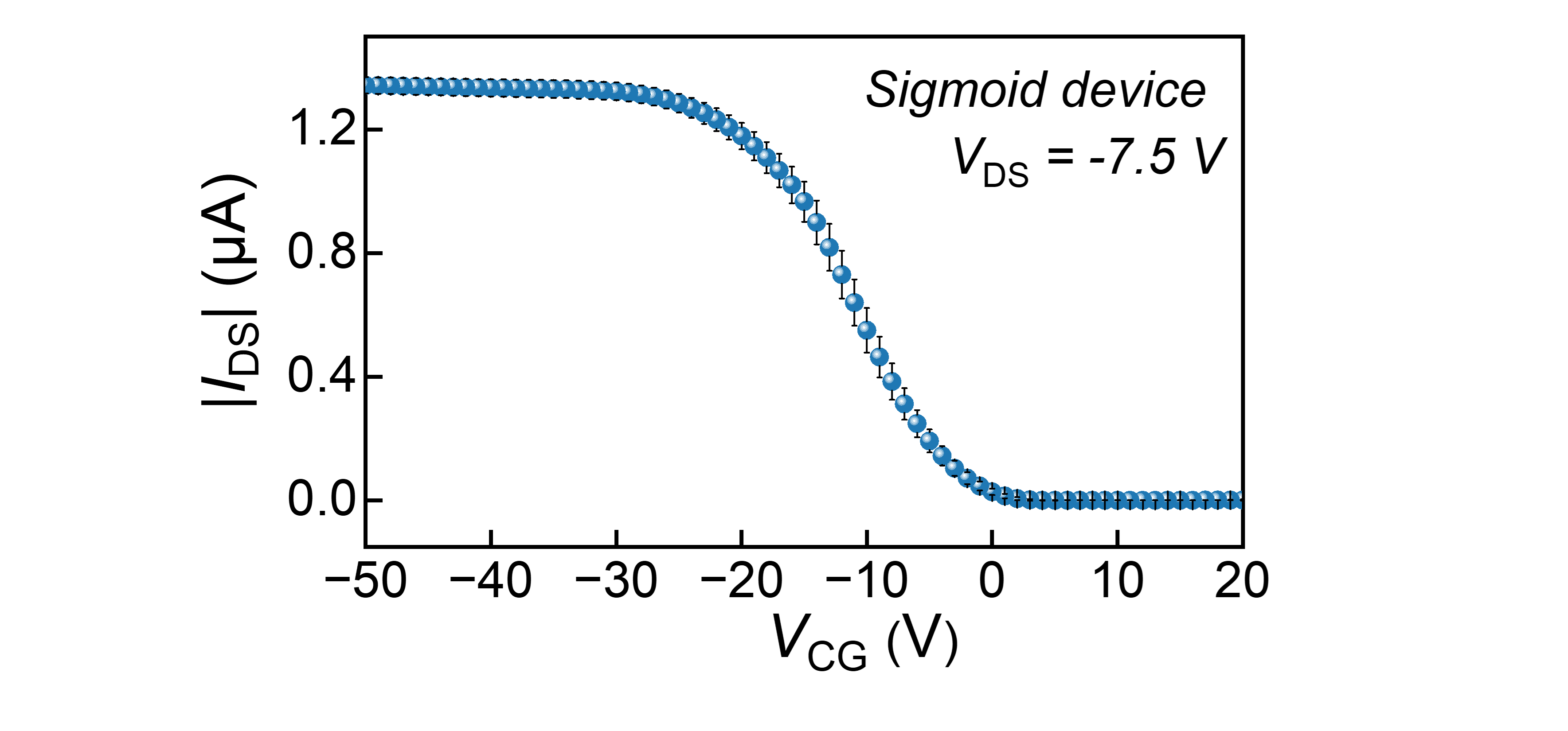


**Figure S34.** Transfer curves (*I*_DS_ versus *V*_CG_) for 50 measured Gaussian activation devices, showing the mean curve and error bars representing the standard deviation across devices.
